# Active-site plasticity revealed in the asymmetric dimer of AnPrx6 the 1-Cys peroxiredoxin and molecular chaperone from *Anabaena* sp. PCC 7120

**DOI:** 10.1038/s41598-017-17044-3

**Published:** 2017-12-07

**Authors:** Yogesh Mishra, Michael Hall, Roland Locmelis, Kwangho Nam, Christopher A. G. Söderberg, Patrik Storm, Neha Chaurasia, Lal Chand Rai, Stefan Jansson, Wolfgang P. Schröder, Uwe H. Sauer

**Affiliations:** 1 Department of Chemistry, Umeå University, SE-901 87 Umeå, Sweden; 2 Umeå Plant Science Centre, Department of Plant Physiology, Umeå University, SE-901 87 Umeå, Sweden; 3 Computational Life-Science Cluster, CLiC, Umeå University, SE-901 87 Umeå, Sweden; 4 Department of Chemistry and Biochemistry University of Texas at Arlington, Arlington, TX 76019-0065 USA; 5 MAX IV Laboratory, CoSAXS beamline, Lund University, Fotongatan 2, SE-225 94 Lund, Sweden; 6 Molecular Biology Section, Laboratory of Algal Biology, Centre of Advanced Study in Botany, Institute of Science, Banaras Hindu University, Varanasi, 221005 India; 7 Department of Biotechnology and Bioinformatics, North Eastern Hill University, Shillong, 793022 India; 8 Present Address: Department of Botany, Centre of Advanced Study in Botany, Institute of Science, Banaras Hindu University, Varanasi, 221005 India

## Abstract

Peroxiredoxins (Prxs) are vital regulators of intracellular reactive oxygen species levels in all living organisms. Their activity depends on one or two catalytically active cysteine residues, the peroxidatic Cys (C_P_) and, if present, the resolving Cys (C_R_). A detailed catalytic cycle has been derived for typical 2-Cys Prxs, however, little is known about the catalytic cycle of 1-Cys Prxs. We have characterized Prx6 from the cyanobacterium *Anabaena* sp. strain PCC7120 (AnPrx6) and found that in addition to the expected peroxidase activity, AnPrx6 can act as a molecular chaperone in its dimeric state, contrary to other Prxs. The AnPrx6 crystal structure at 2.3 Å resolution reveals different active site conformations in each monomer of the asymmetric obligate homo-dimer. Molecular dynamic simulations support the observed structural plasticity. A FSH motif, conserved in 1-Cys Prxs, precedes the active site PxxxTxxCp signature and might contribute to the 1-Cys Prx reaction cycle.

## Introduction

Peroxiredoxins (Prxs) are an essential class of peroxidases (EC 1.11.1.15) ubiquitously found in all known species^1,2^. They efficiently reduce a variety of peroxides, are vital regulators of reactive oxygen species (ROS) levels and play important roles in cell signalling^3–6^. The strictly conserved active site peroxidatic cysteine residue (C_P_) is often located between residues 40 to 60^3^. A common Prx classification is based on the presence and location of a second, the so called resolving cysteine residue (C_R_). The 1-Cys Prxs contain C_P_, but not C_R_, whereas the 2-Cys Prxs have both C_P_ and C_R_. 2-Cys Prxs are further divided into the “atypical” and the “typical” 2-Cys Prxs, depending on whether C_R_ is found in a central position or closer to the C-terminus, and also whether it forms an inter- or intra-molecular disulphide bond with C_P_ during the reaction cycle^3^. Recently, a classification based on Deacon Active Site Profiling (DASP) was introduced by Nelson *et al*.^7,8^ that divides the Prx proteins into six subfamilies: (I) the Prx1/AhpC (“typical” 2-Cys Prxs) and (II) the Prx6 (mainly 1-Cys Prxs) subfamilies, form dimers via the “B” interface through β-strand interactions, whereas (III) the Prx5 (includes “atypical” 2-Cys Prxs) and (IV) Tpx (bacterial periplasmic thiol peroxidase, also includes “atypical” 2-Cys Prxs) subfamilies form dimers across the “A” interface (“A” for alternative or ancestral), (V) the members of the PrxQ subfamily (bacterioferritin co-migrating protein, BCP, and plant PrxQ; includes “atypical” 2-Cys Prxs) remain monomeric, and last (VI) a small subfamily called AhpE (for *Mycobacterium tuberculosis* AhpE). The DASP classification as implemented in the PREX database^8,9^ will be used throughout the text.

The first reference to a Prx was made in 1988 when Kim *et al*. described a thiol specific antioxidant (TSA) protein in *Saccharomyces cerevisiae* that acted as a “Protector Protein”^10^. They then identified a mammalian homologue in the brain of rats with a comparable protective function^11^. Since then, Prx have been associated with multiple metabolic pathways and were linked to a diverse range of processes such as scavenging of ROS and peroxides^12–14^, cell signalling^5^, inflammation, cancer and innate immunity^15–17^, apoptosis^18^, virulence^19^, and colonization^20^ in aerobic and anaerobic bacteria^12,13,19,20^. In addition, the oxidation state of Prx proteins is tightly linked to circadian rhythms in all domains of life^21^. Moreover, unlike other Prx proteins, members of the Prx6 family were shown to carry the additional Ca^2+^-independent phospholipase A2 (PLA_2_) activity^22,23^, reviewed by Fisher^24^.

Peroxiredoxins from the Prx1/AhpC and Prx6 subclasses assemble into antiparallel, obligate (α_2_) homo-dimers via the B-type dimer interface by fusing their central monomer β-sheet into one continuous β-sheet spanning the dimer^25^. The Prx1/AhpC and Prx6 members with two redox active cysteines, can further oligomerize into ring shaped pentamers of dimers (α_2_)_5_, when exposed to oxidative stress or increasing levels of heat. The ring formation is accompanied by a functional switch whereby the proteins lose peroxidase activity and gain molecular chaperone activity^26–30^. Decameric rings were observed, among others, in yeast^27^, *Helicobacter pylori*^28^, *Schistosoma mansoni*^30^ and *Homo sapiens*^31,32^.

Cyanobacteria are ancient and ubiquitous oxygenic prokaryotes^33^ and their Prxs have been studied to some extend (for reviews see^6,7^). Perez-Perez *et al*. suggested that the five different peroxiredoxins found in *Synechocystis* sp. PCC 6803 have additional functions beyond the peroxide detoxification activity^34^. Chaperone activity could be one such function. Dekker *et al*. support the hypothesis that the origin of the Ur-Chaperonin is derived from a thioredoxin/peroxiredoxin fold^35^. Until now however, there has been no report on the three-dimensional structure or functional analysis of a cyanobacterial Prx.

Using two-dimensional gel electrophoresis Mishra *et al*. found that under heat stress a particular protein accumulated in cyanobacteria, which was identified to be AhpC using mass spectrometry^36^. Increased expression of the *Anabaena* sp. PCC7120 AhpC protein (hereafter referred to as AnPrx6) in *E. coli* conferred enhanced resistance to a variety of abiotic stresses, suggesting that AnPrx6 plays an important role in the regulation of cellular processes in addition to the enzymes’ peroxidase activity^37^. These findings and the observation that AnPrx6 contains a sequence motif highly conserved in Prx6 proteins not found in other Prx families, raised the questions: (*i*) does AnPrx6 possess unique functional attributes, and (*ii*) can the high-resolution crystal structure provide a molecular level understanding of the function of AnPrx6 and perhaps of Prx6 proteins in general?

To address these questions, we have crystallized AnPrx6^38^ and present here the first three-dimensional structure of a cyanobacterial Prx6 protein together with results obtained from bioinformatics analysis and from molecular dynamics simulations. Further, biochemical analyses provide evidence that AnPrx6 is unique among the Prx proteins, since it can act as a molecular chaperone in its dimeric state, contrary to other Prxs.

## Results

### Sequence analysis

Multiple sequence alignments (MSA) of Prx proteins and phylogenetic analysis with Clustal Omega^39^ established AnPrx6 as a member of the Prx6 subfamily. A BLASTp search^40^ with AnPrx6 as query against the Protein Data Bank (PDB)^41^ shows that the human Prx6 protein hORF6 (PDB code 1PRX^42^) shares 52% sequence identity with AnPrx6 and is the highest scoring orthologue with a BLASTp bits score of 544. hORF6 is the founding member of the Prx6 subfamily^25^ and carries a single cysteine residue, similar to AnPrx6. Additional high scoring orthologues include Prx6 from *Arenicola marina* (PDB code 2V2G^43^) and Prx6 protein from *Plasmodium yoelii* (PDB code 1XCC^44^) with 48% and 41% sequence identity, respectively. The sequence identity shared with two members of the Prx6 family that form (α_2_)_5_ decamer rings is slightly lower: 39% for the protein from the aerobic hyperthermophilic Crenarchaeon *Aeropyrum pernix* K1 (PDB code 2CV4^45^) and 38% for the protein from the anaerobic hyperthermophilic archaeon *Pyrococcus horikoshii* (PDB code 3W6G^46^). Sequence similarities and differences are further discussed in the context of structural comparisons.

### X-ray crystal structure determination and analysis

The crystal structure of AnPrx6 was determined from orthorhombic crystals in space group P2_1_2_1_2_1_^38^. Phase information and an initial atomic model were obtained by molecular replacement (MR) using the monomer A of human Prx6 (PDB code 1PRX^42^), as the search model. After multiple rounds of model building and refinement against data extending to a d-spacing of 2.3 Å the values for R_work_ and R_free_ converged at 20.4% and 24.5%, respectively. (For details see Supplementary Table S1). The crystallographic asymmetric unit contains four copies of the AnPrx6 protein, monomers A to D. In the case of monomers A and C, all native amino acids (Met1 to Lys212) could be built into the electron density map, whereas monomers B and D lack the N-terminal methionine. The four monomers associate into two dimers, AB and CD. Superimposition of the peptide backbone atoms of dimer AB onto dimer CD results in a low root mean square deviation (RMSD) of 0.22 Å which means that the two dimers are basically identical. We therefore refer to dimer AB in all further discussions, unless indicated otherwise.

### The AnPrx6 monomers and the asymmetric dimer

**Figure 1:**
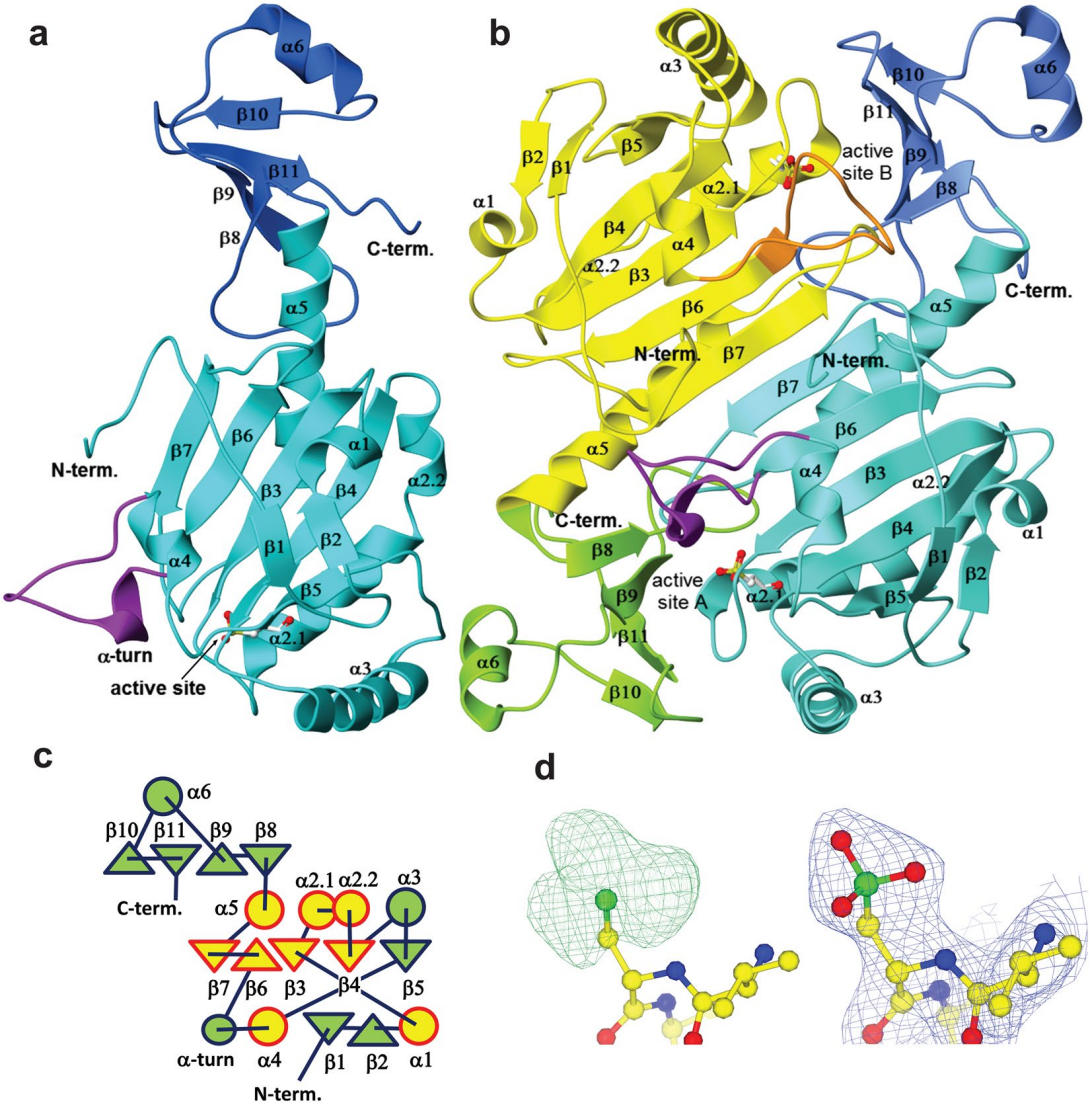
The Crystal structure of AnPrx6. (**a**) Ribbon diagram of monomer A. The catalytic N-terminal peroxiredoxin domain (M1-N162) is coloured light blue and the C-terminal dimerization domain (Y163-K212) dark blue. The termini and secondary structure elements are labelled. The active site C_P_ is shown  in ball-and-stick style. (**b**) Ribbon diagram of the AnPrx6 dimer. Monomer A is coloured as before. The N-terminal domain of monomer B is shown in yellow and the C-terminal domain in light green. The residues linking helix α4 to strand β6 fold into an α-helical turn in monomers A and C (purple) while they adopt an irregular loop in monomers B and D (orange). (**c**) Topology diagram of AnPrx6. The core secondary structure elements of the Trx fold are highlighted in yellow and red. Note that the α-helical turn is only present in AnPrx6 monomers A and C. (**d**) Left panel: A positive feature in the difference electron density map (*m*|F_o_| − D|F_c_|), calculated with a Cys at the active site and contoured at 3 RMSD, arises due to excess electrons (green mesh) covering the sulphur atom (green ball). This suggests that the active site Cys45 (C_P_) is triple oxidized to cysteine-sulfonic acid (Cys-O_2_-OH). Right panel: The quality of the (2 *m*|F_o_| − D|F_c_|) electron density map, contoured at 1 RMSD (blue mesh), after structure refinement with cysteine-sulfonic acid at position 45. All atoms and their electrons are accounted for and no extra difference electron density features are visible in the map. (|F_o_| and |F_c_| are the modulus of the observed and calculated structure factors, respectively, *m* is a figure of merit and *D* is a scale factor).

The overall structure of an AnPrx6 monomer (Fig. 1a) and dimer (Fig. 1b) is similar to known structures of 1-Cys and 2-Cys Prxs from the Prx1/AhpC and Prx6 subfamilies. Each monomer consists of two domains: an N-terminal peroxiredoxin domain (residues Met1 to Asn162) and a C-terminal dimerization domain (residues Tyr163 to Lys212).

The N-terminal catalytic domain comprises seven β-strands and five α-helices. Five strands (β7, β6, β3, β4, and β5, from left to right in monomer A, Fig. 1a) assemble into the central β-sheet that is sandwiched between a β-hairpin, formed by strands β1 and β2, and helices α1 and α4 on one face and helices α2, α3 and α5 on the opposite side. The core of the N-terminal domain corresponds to the conserved thioredoxin fold^1,25^, consisting of beta strands β7, β6, β3 and β4 and α-helices α1, α4, α2 and α5 (highlighted in yellow and red in the topology diagram of Fig. 1c). Helix α2 contains a kink introduced at Pro57 that divides α2 into two sections denoted α2.1 and α2.2. The first turn of helix α2.1 contains the strictly conserved peroxidatic cysteine (C_P_). In this structure, the sulphur atom of C_P_ (Cys45) is triple oxidized to cysteine-sulfonic acid (Cys45-O_2_-OH), which is evident from three additional features in the (m|F_o_| - D|F_c_|) difference electron density map (Fig. 1d).

The C-terminal dimerization domain folds into a four stranded anti-parallel β-sheet formed by strands β8, β9, β10 and β11 and a helix, α6, located between strands β9 and β10, that caps the sheet. The dimerization domain stabilizes the assembly of two monomers into an antiparallel obligate domain-swapped dimer as illustrated in Fig. 1b.

In all Prx6 structures determined so far, the monomers constituting the dimers are almost identical, with RMSD values below 0.5 Å and frequently below 0.3 Å. This is not the case for AnPrx6. Superimposing monomer A onto B and C onto D results in RMSD values of 0.87 Å and 0.94 Å, respectively, which is due to differences distributed throughout the two monomers. However, superimposing monomers A onto C and monomer B onto D results in almost perfect matches with RMSD values of about 0.2 Å in both cases.

In order to place the AnPrx6 protein into a greater structural context, its structure was simultaneously superimposed with thirteen representative Prx structures and a structure anchored multiple sequence alignment (saMSA) was obtained (Supplementary Fig. S1). The saMSA shows that residues Pro38, Thr42 and Cys45 of AnPrx6 map to the invariant Prx active site pattern P-X(3)-[TS]-X(2)-C, where Ser is observed in rare cases, X marks any amino acid and C is the peroxidatic Cys (C_P_). In addition, Arg122 matches the strictly conserved active site arginine found in all known peroxiredoxins.

Although the crystal structures of the fourteen proteins are mutually similar, with RMSD values ranging between 1.1 Å and 1.7 Å, they clearly differ in several key regions. One such region involves helix α3 and the loop connecting α3 with strand β5 that varies both in length and conformation among Prx6 family members and is distinctly different compared to the corresponding region of (α_2_)_5_ decameric ring forming Prxs, where helix α3 is approximately two turns shorter and the α3-β5 loop can form a 3_10_ helical turn. (Supplementary Fig. S2).

**Figure 2:**
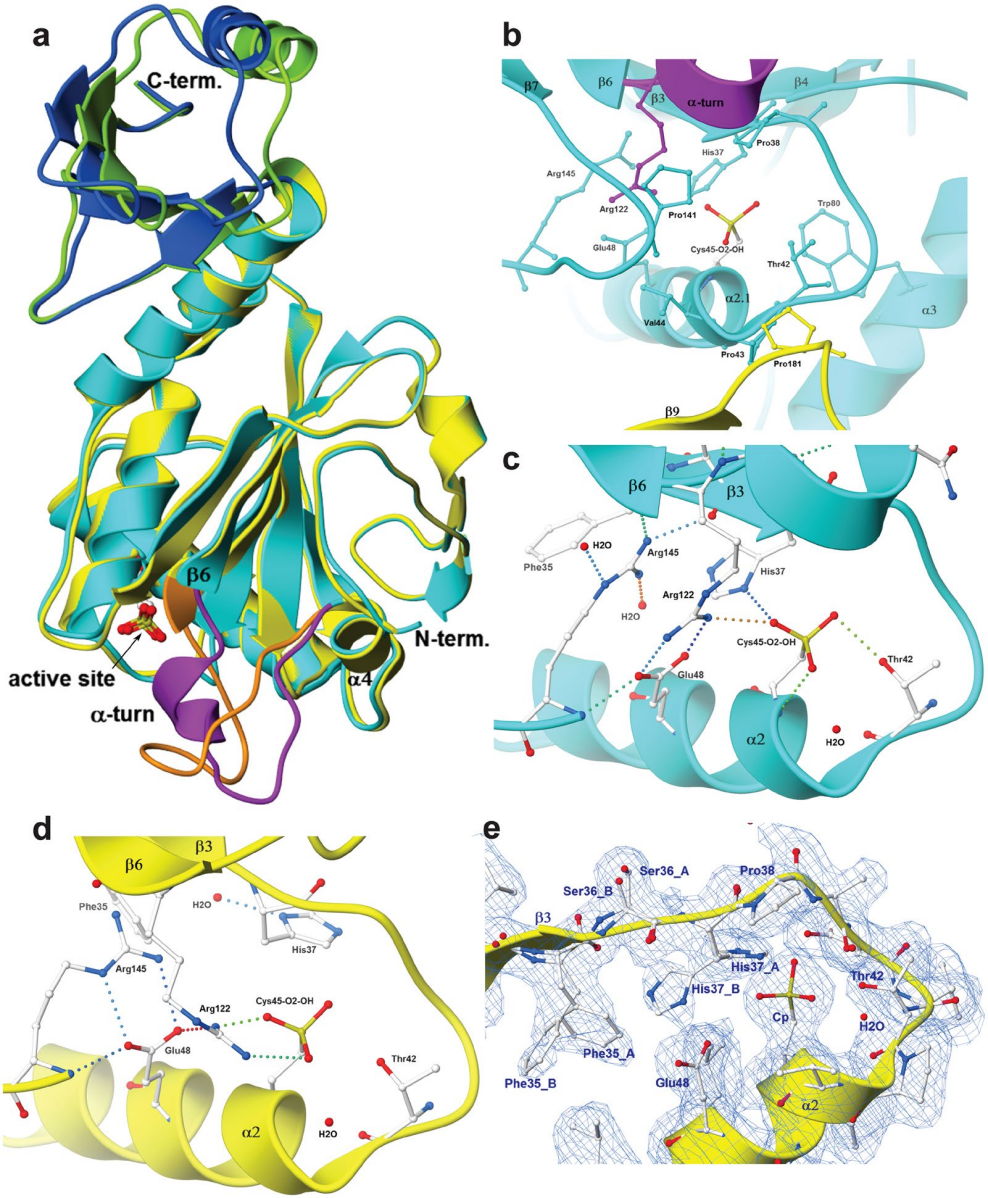
The asymmetric monomers. (**a**) Superimposition of monomer A (light and dark blue) and B (yellow and green) highlights their overall conformational differences especially the loop connecting α4 and β6 (Asp109 to the conserved Arg122). Residues Ala117 to Thr120 of monomer A fold into an α-helical turn, while they form an irregular loop in monomer B, thus affecting the geometry of the active site. (**b**) View of the residues lining the active site environment in monomer A. The α-helical turn is oriented such that the carbonyl oxygen atoms and the helical axis point towards C_P_. Part of strand β9 and Pro181 from monomer B are marked in yellow. (**c**) Hydrogen bonding network of monomer A and monomer C. The Cys45-O_2_-OH forms a H-bond (dotted line) through its Oδ2 atom with the oxygen (Oγ1) of Thr42, and through its Oδ1 atom an H-bond with the Nδ1 atom of His37. While Arg122 mainly engages in a salt-bridge with Glu48, it also forms a H-bond through its Nη1 to the Oδ1 of Cys-O_2_-OH. Downstream in the sequence but close in space lies Arg145 whose main chain nitrogen atom H-bonds to the Oε1 atom of Glu48 and its Nη2 atom forms a bifurcated H-bond with the carbonyl oxygen atoms of Ser36 and Arg122. In addition, the Nε and Nη1 of Arg145 bind one water molecule each. A third water molecule is held in place by the main-chain nitrogen atom of Val44 and does not directly H-bond with Cys45-O_2_-OH. A corresponding water molecule is found in many Prx structures. (**d**) The active site of monomer D. Here, the side chain of His37 flips away from C_P_ and forms an H-bond with a water molecule. Arg122 forms a salt bridge with the oxygen atoms Oδ1 and Oδ3 of Cys45-O_2_-OH, and maintains a H-bond to Glu48. The side chain of Arg145 rotates into a position where its Nε and Nη2 atoms can form a new salt bridge with the Oε1 and Oε2 atoms of Glu48. The rearrangement leads to a shift of the C_P_-loop, which disrupts the H-bond between C_P_ and Thr42. (**e**) The electron density (blue mesh) covering the active site residues of monomer B clearly suggests alternate conformations for Phe35, Ser36 and His37, which reflects the dynamics of these residues.

The second region of striking structural variability affects the amino acids between Asp109 and the conserved Arg122 that connect helix α4 with strand β6. This region is highlighted in purple and orange in monomer A and B, respectively in Fig. 1a,b and Supplementary Fig. S2. In some Prx proteins this region adopts an extended, irregular loop, e.g. in monomers B and D of AnPrx6; in hORF6 (PDB entry 1PRX^42^) and in *Aeropyrum pernix* Prx6 (PDB entry 2CV4^45^), whereas it folds into a short two stranded β-sheet in others, e.g. *Arenicola marina* Prx6 (PDB entry 2V2G^43^) and *Trypanosoma cruzi*, Prx1 (PDB entry 4LLR^47^). In contrast, residues Ala117 to Thr120 of AnPrx6 monomers A and C fold into an α-helical turn (marked purple in Fig. 1a,b), while the same region of monomers B and D forms an extended loop (orange in Figs 1b, 2a and Supplementary Fig. S2). The α-helical turn is a novel feature which was not previously observed in any Prx structure. Additional minor structural variations propagate throughout the monomers A and B and become visible as colour differences between the light blue and yellow ribbons (Fig. 2a). The differences are emphasized in loop regions and are clearly visible in the displacement of the C-terminal domains.

### Conformational asymmetry of the active site environment

The helix α2 contains the peroxidatic cysteine (C_P_) and is part of the β3-α2-β4 motif, common to all Prx proteins. The strictly conserved residues Pro38 and Thr42 of the catalytic PxxxTxxC_P_ motif are part of the loop connecting β3 with α2. The so-called region I and the C_P_-loop^26^ overlap with this loop and continue into the first two turns of helix α2. The C_P_-loop adopts a “fully folded” (FF) conformation^4,25^ in all four AnPrx6 monomers, thereby positioning C_P_ at the bottom of the active site pocket bounded by residues Phe35, His37, Pro38, Thr42, Pro43, Val44, Glu48, Trp80, Arg122, Pro141 and Arg145 of the N-terminal domain and Pro181 from the C-terminal domain of the adjacent monomer (Fig. 2b). The histidine residue directly preceding the β3-α2 loop (His37 in AnPrx6) is conserved in all Prx6 family members, while 2-Cys Prx proteins contain a Tyr or, in rare cases, a Trp at the corresponding position.

Despite apparently identical C_P_ oxidation states in all four monomers, the positions and hydrogen bonding patterns of key active site residues, including Phe35, His37, Cys45-O2-OH (C_P_), Arg122 and Arg145, differ in the two monomers within the dimer. In monomer A and similarly in monomer C, the oxygen atom Oδ1 of Cys45-O_2_-OH forms hydrogen bonds with the Nδ1 atom of His37 and the Nη1 atom of the strictly conserved Arg122 (Fig. 2c). Moreover, the guanidinium group of Arg122 forms a salt-bridge with the side chain carboxylic acid of Glu48. The guanidinium group of the conserved Arg145 participates in a hydrogen bond network that includes the carboxyl oxygen atoms of Ser36 and Arg122 as well as two water molecules that are bound to its Nη1 and Nε atoms. In monomer A, Arg145 interacts through the main chain N atom with Glu48, which is positioned one turn away from C_P_ on helix α2.1. An active site water molecule, which is coordinated by the main chain N atom of Val44, is present in all four AnPrx6 monomers and aligns almost linearly with Sγ of C_P_ and Nδ1 of His37. It was proposed for the human hORF6 protein (PDB code 1PRX^42^) that an equivalent water molecule participates in the Prx6 reaction mechanism^42^. The arrangement of the active site residues, the water molecules and hydrogen bonds observed in monomers A and C of AnPrx6 are nearly identical to the side chain orientations and H-bond patterns found in the human hORF6 protein (PDB code 1PRX^42^) and in the active site arrangement of the hypervalent sulphur intermediate of *Aeropyrum pernix* K1 (PDB code 2ZCT^48^) where only Phe40 deviates from the orientation of Phe35 in monomer A of AnPrx6.

Close inspection of the active site residues of monomer D (Fig. 2d), shows variations compared to monomer B, as discussed in the next paragraph, and clear differences in orientation compared to monomer A. Conformational changes affect the peptide backbone, the side chain positions and, consequently, the H-bond pattern. The most striking difference is the placement of His37, whose side chain dihedral angle changed by about −130° (clockwise rotation), compared to its orientation in monomer A. His37 adopts a position away from C_P_, where its Nδ1 atom forms a hydrogen bond with a water molecule. The new His37 orientation becomes possible due to a movement of strand β4 and the following loop, which allows a counter clock wise rotation of the Oγ of Ser70 thus providing a space for His37. Due to the formation of an α-helical turn in the loop connecting α4 to β6 of monomer A, Arg122 rotates away from Glu48 and engages in a salt-bridge interaction with the Oδ1 and Oδ3 of the triple oxidized Cys45. Being released from the interaction with Arg122, Glu48 forms a new salt-bridge with the Nε and Nη2 atoms of Arg145. It is noteworthy that Phe35 and Ser36 follow the move of His37. Especially the side chain dihedral angle of Phe35 changes by about −65° (clockwise rotation) and the Oγ of Ser36 rotates about + 125° (counter clockwise) compared to monomer A, respectively. The three residues form the FSH motif that is strictly conserved in all known Prx6 proteins. It is striking that the active site arrangement of amino acids in monomer D is almost identical to the active site of the reduced (SH) and the sulfinic acid (SO_2_H) form of human Prx6 (PDB codes 5B6M^49^ and 5B6N^49^) with an overall RMSD of 0.42 Å to both structures. Likewise, the active site of monomer D is reminiscent of the active sites of *Plasmodium yoelii* Prx6 (PDB code 1XCC^44^), *Arenicola marina* Prx6 (PDB code 2V2G^43^) as well as the fully folded active sites of the decameric 2-Cys peroxiredoxins from Human Erythrocytes (PDB code 1QMV^50^) and *Schistosoma mansoni* (PDB code 3ZTL^30^). In addition, the orientation of active site residues in monomer D match even the corresponding amino acid arrangement found in 2-Cys Prxs with locally unfolded C_P_-loops, except for the position of C_P_ itself (1E2Y^51^, 3TUE^52^, 4FH8^53^).

Unexpectedly, the electron density surrounding the active site of monomer B (Fig. 2e) suggests two clearly discernible alternative conformations for Phe35, and His37, while the two conformations of Ser36 are both comparable to the one observed in monomer D. The orientations of Phe35 and His37 assigned to the stronger electron density were refined to an occupancy of about 0.6, and the occupancy for the second orientation, corresponding to the weaker electron density, amounted to 0.4. The positions of Phe35 and His37 with the higher occupancy match those observed in monomer D, where His37 is pointing away from C_P_. The positions with the lower occupancy are similar but not identical to those of Phe35 and His37 observed in monomer A and C. The crystallographic data alone do not provide a conclusive answer as to whether the alternate positions were due to dynamic motions or due to distinct static positions. In case of dynamic motion, it is conceivable that the movements of the FSH motif residues, Phe35, Ser36 and His37, are correlated. It is also possible that the plasticity of the peptide main chain, including the formation of the helical turn are necessary for the movements of Phe35, Ser36, His37, Arg122 and Arg145 and hence for the catalytic activity of AnPrx6.

The fact that the His37 directly precedes the C_P_-loop and is strictly conserved within the Prx6 subfamily, suggests an important role in the catalytic cycle of Prx6 proteins. It has been suggested that the conserved histidine together with a strictly conserved arginine residue (His37 and Arg122 in AnPrx6) facilitate the deprotonation of C_P_ by lowering its pK_a_ and stabilizing the thiolate form, which is essential for catalysis^42,54,55^. Despite the accumulated knowledge, the exact catalytic cycle for AnPrx6 is not yet known and to which extend Phe35, Ser36 and His37 are involved will be the focus of further studies. To date, no single electron donor has been conclusively identified for the reduction of the oxidized C_P_ in members of the 1-Cys Prx6 subfamily. Various suggestions were put forward on how the oxidized C_P_ is returned to the reduced state. The protected location of C_P_ within the environment of the active site pocket seemingly prevents its reduction by a protein agent such as thioredoxin. Instead, various small reducing agents, such as glutathione (GSH)^24,56^ and ascorbate (vitamin C)^57^, have been suggested.

### Molecular dynamics simulations

**Figure 3:**
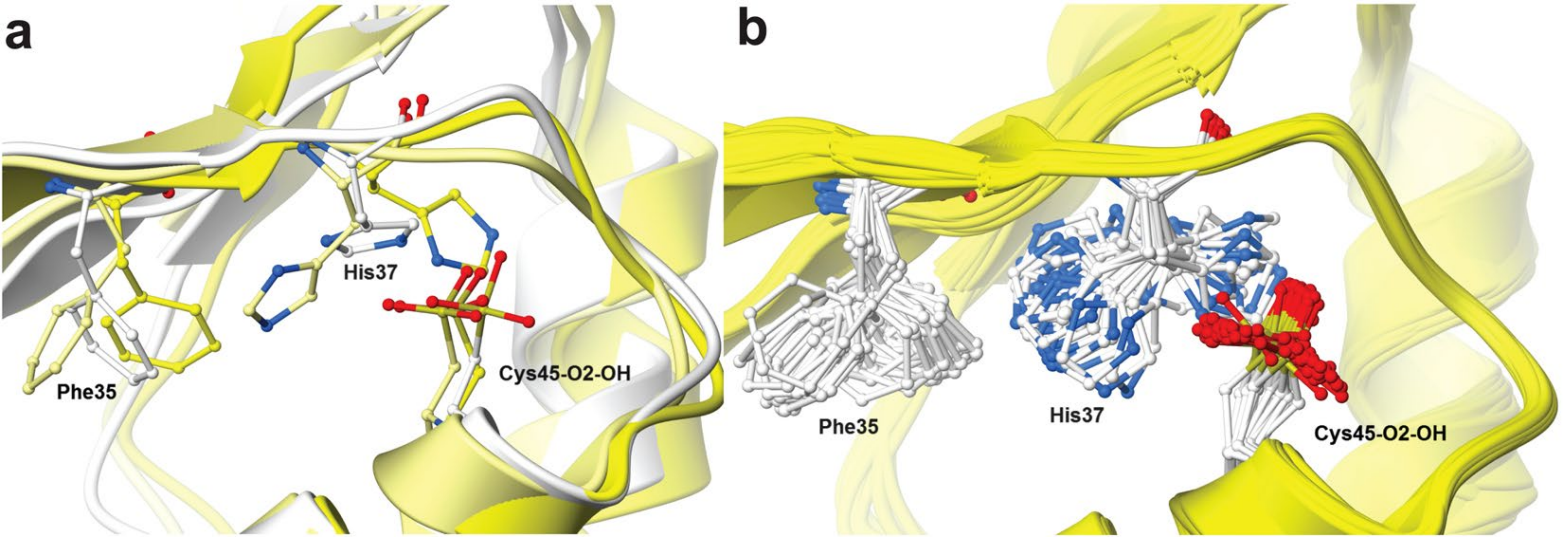
Visualisation of the dynamic movement of Phe35 and His37. (**a**) The active site of monomer B at three different time points along the MD trajectory (t = 1 ns, white, 20 ns, light yellow and 75 ns, yellow) visualizes the coordinated motion of Phe35 and His37. The His37 side chain dihedral angle of about −180° corresponds to the conformation found in monomer A and the value of −57° to that found in monomer D. The active site C_P_ (Cys45-O_2_-OH) does not move significantly. (**b**) Active site of monomer B after Ensemble Refinement. The final Ensemble contained 45 models of the AnPrx6 structure. While Phe35 and His37 display multiple alternative orientations, the orientation of the oxidized C_P_ does not vary significantly. Although the MD simulations and the Ensemble refinement are independent methods, they both suggest large conformational oscillations for His37 and Phe35 in monomer B.

To test the hypothesis of dynamic, correlated movement of Phe35 and His37 and to examine whether the asymmetry of the AnPrx6 dimer observed in the crystal is a feature maintained in solution, molecular dynamics (MD) simulations were performed. The isolated AnPrx6 dimer (monomers A and B) was surrounded by a periodic boundary box of water molecules and simulated for 150 ns. To confirm the simulation results, the simulations were performed in triplicate, starting from different velocity distributions. The MD trajectory describes a dynamic dimer and indicates that the structural features of each monomer (i.e., the α-helical turn between α4 and β6 in monomer A and the extended loop in the same region of monomer B) can fluctuate around the positions observed in the X-ray structure throughout the simulation (Supplementary Fig. S3). The results imply that the asymmetry is a feature of the dimer which is maintained in solution and is not due to crystal packing interactions. The MD simulations also showed the correlated interconversion of the side chain orientations of Phe35 and His37 in monomer B (Fig. 3a, Supplementary Fig. S3 and the Supplementary simulations Sim1 and Sim3, corresponding to Simulation [1] and Simulation [3] in Supplementary Fig. S3e), whereas the side chains of the two residues in monomer A remained close to their positions observed in the X-ray structure (Simulation [2] in Supplementary Fig. S3e). The variations of the His37-Cys45 and Arg122-Cys45 distances are different for monomer A and B (Supplementary Fig. S4). Monomer A shows larger distance fluctuations and longer Arg122-Cys45 H-bond distances compared to those of monomer B, whereas in monomer B the His37-Cys45 distance fluctuations are more pronounced and the H-bond distances are longer compared to monomer A. The opposite trend between the two H-bond interactions might occur because the Arg122-Cys45 interaction is strong (or more stable) in monomer B, and allows His37 to be released from Cys45 and move more freely. In contrast, in monomer A, as Arg122 interacts weakly with Cys45, Cys45 interacts more strongly with His37, thus limiting its motion. This difference suggests a correlation between the two C_P_ interactions and the increased mobility of His37 (via concerted motion with Phe35).

Taken together the MD simulations strengthen the hypothesis that the α-helical turn between α4 and β6 in monomer A (and C) leads to a more restricted and hence more ordered active site, as depicted in Fig. 2c, where His37 is hydrogen bonded to the oxidized C_P_. The simulations further indicate that the unstructured loop connecting α4 and β6 in monomer B facilitates the correlated movement of Phe35 and His37 and, while His37 is pointing away from C_P_, Arg122 forms a salt bridge with the active site cysteine-sulfonic acid.

### Synergistic effect upon dimer formation

Two AnPrx6 monomers associate into an asymmetric, antiparallel obligate homo-dimer. To highlight the conformational asymmetry between the two monomers, we refer to the dimer as an (αα*) type homo-dimer, in order to distinguish it from commonly observed symmetric (α_2_) type homo-dimers. The AnPrx6 monomers interact through hydrogen bonds across an extended interface. The central contacts are formed through four main-chain hydrogen bonds across the B-type dimer interface, involving Thr136 and Thr138 of strands β7 from each monomer. The dimer is further stabilized through a hydrogen bond network created by main- and side chain hydrogen bonds of residues from the loop between β7 and α5 (Tyr139, Ser142, Thr143, Gly144, Arg145) as well as residues from α5 (Phe147, Glu149, R152). Additional interfacial H-bond contacts are provided by residues from the “domain swapped” C-terminal domains (amino acids Tyr163 to Lys212). Specifically, residues from the loop connecting β8 and β9 (Thr167, Ala169, Asp170) of one monomer form hydrogen bonds with residues of the loop linking β7 and α5 (Tyr139, Ser142, Gly144, Asn146) from the second monomer and with residues T47 on helix α2.2. Even Lys201 and Tyr203 of the C-terminal loop connecting β10 and β11 interact with Asp83 and Glu86, respectively, from α3 of the second monomer. Finally, Arg4 of the N-terminal tail of one monomer interacts with the backbone oxygen of Asp109 from the other monomer. Taken together, these hydrogen bonds are part of the extended dimerization interface that buries about 2185 Å^2^ of the surface area from monomer A and 2199 Å^2^ from monomer B, as determined by PDBePISA^58^, which amounts for approximately 21% of the total surface area of each monomer.

### AnPrx6 does not form higher molecular weight oligomers

Members of the Prx1/AhpC and some members of the Prx6 family are able to assemble into (α_2_)_5_ decameric rings in response to increasing oxidative stresses^25,26^. To experimentally verify the oligomeric state of AnPrx6 in solution, analytical size exclusion chromatography (SEC) analysis was carried out at three different protein concentrations (0.1 mg ml^−1^, 1 mg ml^−1^ and 10 mg ml^−1^) in the absence or presence of 20 mM H_2_O_2_. Each chromatogram contained a single retention peak. In the absence of H_2_O_2_ the peak positions were observed at retention volumes of 15.38 ml, 15.34 ml and 15.40 ml. In the presence of 20 mM H_2_O_2_ the retention volumes were 15.53 ml, 15.37 ml and 15.18 ml (Supplementary Fig. S5). Using a standard curve (BioRad Gel Filtration Standard) to convert the retention volumes to molecular weights, one finds that the average molecular weight in the absence of H_2_O_2_ is 44,2 kDa and 44,7 kDa in the presence of 20 ml H_2_O_2_. These values are in good agreement with the calculated value for dimeric AnPrx6 (48.4 kDa).

No peaks were detected at lower retention volumes. In particular there was no peak at a retention volume of 12 ml, which would correspond to the molecular weight of a (α_2_)_5_ decamer (242 kDa). The results from the SEC measurements confirm that AnPrx6 is dimeric in the native, reduced state as well as under oxidative conditions and, unlike the Prx1/AhpC proteins from yeast^27^ and *Helicobacter pylori*^28^, does not undergo oxidative stress-dependent oligomerization.

In the crystallographic asymmetric unit (a.s.u.) of AnPrx6 crystals in space group P2_1_2_1_2_1_, two AnPrx6 dimers pack against each other. They associate through helix α6 of the C-terminal domain, through residues from the N-terminal loop and the loop between helix α4 and strand β6, thereby burying an interface of 690 Å^2^ (PDBePISA^58^) (Supplementary Fig. S6). Considering the small size of the contact area and that the two dimers are related by a non-crystallographic twofold symmetry axis, one can conclude that the dimer-dimer interaction is not biologically relevant. Further contacts between neighbouring asymmetric units are propagated through strands β10 of the C-terminal extensions (Supplementary Fig. S6) and neither of the packing contacts is mediated through the A-type interface and will not lead to decameric rings.

Small angle X-ray scattering (SAXS) measurements were carried out at two protein concentrations (1.6 mg ml^−1^ and 4.8 mg ml^−1^) to verify whether AnPrx6 exists exclusively in its dimeric state in solution. The resulting SAXS scattering curves coincided and show variation only in the level of noise (Supplementary Fig. S7). From Guinier plot analysis the radius of gyration was determined to be R_g_ = 2.98 ± 0.03 nm for both concentrations. This value corresponds to a hydrodynamic diameter 2r ≈ 7.7 nm, which is in good agreement with the X-ray structure, where the largest distance within the AnPrx6 dimer is approximately 8,1 nm. The molecular weight of the scattering particles in solution was estimated to be 44 kDa, using Hen Egg White Lysozyme as the molecular weight standard. Similarly, using the Porod volume, the molecular weight was estimated to be 48 kDa. Considering that the calculated molecular weight of the AnPrx6 monomer is 24.2 kDa, one can conclude from the SAXS data that AnPrx6 is a dimer in solution.

### AnPrx6 acts both as a peroxidase and as molecular chaperone

#### Peroxidase activity

Enzymatic peroxidase activity of AnPrx6 was determined according to the FOX assay as described in Jiang *et al*.^59^ with hydrogen peroxide (H_2_O_2_) as the substrate. The enzyme was incubated in 600 μM H_2_O_2_ and the reaction was terminated after one minute by addition of FOX reagent. Based on the observed absorption at 560 nm, the H_2_O_2_ consumption can be determined from a standard curve and the peroxidase activity of AnPrx6 was calculated to be 230 ± 12 nmol H_2_O_2_ min^−1^ (mg of AnPrx6)^−1^. These results suggest that the overexpressed and purified AnPrx6 protein has a moderate peroxidase activity as compared to PrxQ that showed 30 times higher activity measured under the same conditions. In order to assess if the low peroxidase activity could be due to over-oxidation of the enzyme, the activity was measured at increasing concentrations of H_2_O_2_, starting from 0 to 600 μM H_2_O_2_. The peroxidase activity increased almost linearly up to 100 μM H_2_O_2_, where it levelled out at 230 ± 12 nmol H_2_O_2_ min^−1^ (mg of AnPrx6)^−1^ (Supplementary Fig. S8). This suggests that the peroxidase activity is saturated at approximately 100 µM H_2_O_2_ and that the enzyme is able to function also at higher H_2_O_2_ concentrations under incubation times less than 1 minute. Furthermore, a linear dependency of the rate of H_2_O_2_ consumption was observed when increasing the AnPrx6 concentrations between 0 μM to 60 μM and using a fixed, saturating amount H_2_O_2_ (Supplementary Fig. S9).

#### Chaperone activity

**Figure 4:**
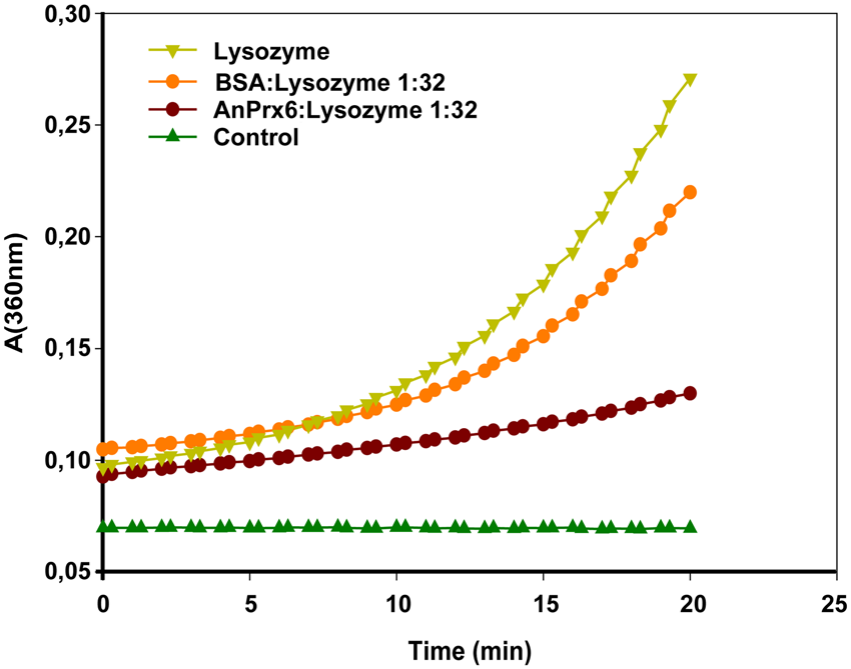
Chaperone activity assay. Thermal aggregation of HEWL at 43 °C was monitored at a wavelength of 360 nm by measuring the degree of light scattering at different time points. The reference level of scattering was determined with a reaction mixture containing only buffer, HEWL and DTT (light green triangles). Aggregation is strongly reduced in the presence of dilute AnPrx6 (brown dots), whereas the same dilution of BSA (positive control) has almost no effect on aggregation (orange dots). Buffer and DTT (dark green triangles) were used as negative control (blank).

Chaperone activity for 1-Cys Prx6 proteins has not yet been reported. In order to enquire potential chaperone activity of AnPrx6, a chaperone assay that uses Hen Egg White Lysozyme (HEWL) as substrate was employed^60^. Lysozyme is a monomeric protein of 14.6 kDa with four disulphide bonds stabilizing its tertiary structure. Reduction of a lysozyme solution with dithiothreitol (DTT) and heating at 43 °C causes it to partially unfold, which leads to aggregation and precipitation and results in increased light scattering. The thermal aggregation of HEWL at 43 °C in the presence of DTT and AnPrx6 or BSA was monitored by measuring the light scattering of the reaction mixture at a wavelength of 360 nm. In control experiments without AnPrx6, the scattering intensity increased exponentially over time, indicating that the rate of aggregation and precipitation increased as the assay proceeded. The addition of AnPrx6 at a molar ratio of 1:32 (AnPrx6 to Lysozyme) inhibited the thermal aggregation of HEWL (Fig. 4). Replacing AnPrx6 with BSA had a slight inhibitory effect on the aggregation of HEWL (Fig. 4). This was expected since BSA is known to possess moderate chaperone activity^61,62^. Additional experiments, where the molar ratio of AnPrx6 to lysozyme was adjusted to 1:22 and 1:16 revealed a chaperone activity independent of the AnPrx6 concentration within the tested range of molar ratios, whereas BSA showed little or no chaperone activity (Supplementary Fig. S10).

## Discussion

The crystal structure of AnPrx6 provides a first glimpse of a cyanobacterial 1-Cys Prx protein and is an unique example of an asymmetric Prx6 homo-dimer. The structural asymmetry within the so called αα* type homo-dimer was confirmed through molecular dynamics simulations, which showed that the motions of Phe35 and His37, of the FSH motif, are correlated. The asymmetry has an impact on the positions and interactions of key active site residues, in particular the placement of His37 and Arg122, which will affect the pK_a_ value of the active site cysteine (C_P_) and hence its reactivity. Remarkably, the two different active site conformations observed within the AnPrx6 dimer, each resemble an active site conformation that had been previously reported for symmetric (α_2_) Prx dimers from different species and with different oxidation states. The asymmetry together with backbone fluctuations around the active site could be a common feature of 1-Cys Prx6 proteins and crucial for their activity.

We propose that, in the particular case of AnPrx6 and perhaps the Prx6 subclass, the asymmetric structural fluctuations propagate through the dimer and accompany or possibly induce the movement of the conserved FSH motif and particularly the conserved His37 residue that precedes the C_P_-loop. These fluctuations, together with the movements of Arg122, are likely to contribute to a shift of the pK_a_ value of the peroxidatic C_P_, such that one active site is primed for catalysis while the other can be reduced. Yuan *et al*.^63^ and Salsbury *et al*.^64^ predicted similar asymmetric Prx dimers from MD simulations. However, the AnPrx6 structure is so far the only example of an experimentally observed asymmetry within a Prx6 dimer.

Furthermore, our functional analyses provide evidence that the AnPrx6 homo-dimer harbours dual functions. It acts as a peroxidase and, in its dimeric state as a molecular chaperone. The absence of high molecular weight oligomers was confirmed by SAXS measurements and size exclusion chromatography, the latter carried out under native and H_2_O_2_ stress conditions.

We speculate that AnPrx6 is a unique and ancient member of the Prx family that has been retained in cyanobacteria and might represent an early state of Prxs evolution, capable of both peroxidase and chaperone functions, without the need to change its oligomeric state.

Organisms that perform oxygenic photosynthesis face unique challenges in terms of ROS management and protein folding because photosystem II is both a powerful generator of ROS and requires assembly and frequent regeneration due to the very rapid turnover of the D1 subunit, even under normal physiological conditions^65^. Although the immediate effect of AnPrx6 and other peroxiredoxins on photosystem II is not yet understood^66^ it is possible that the dual functions of AnPrx6 and its unique oligomerization behaviour evolved to cope with these challenges, especially since cyanobacteria frequently inhabit extreme environments and emerged at a time when the earth’s atmosphere contained only a small fraction of oxygen. Indeed, the cyanobacterial photosynthesis was the key driver in changing the planet’s atmosphere from reducing to oxidizing. It is intriguing to speculate that the first adaptations to life under highly oxidizing conditions may have occurred inside the cells of oxygen-producing cyanobacteria and that this may be the origin of the unique characteristics of AnPrx6, a cyanobacterial 1-Cys Prx6.

## Materials and Methods

### Cloning, expression, purification and crystallization of AnPrx6

The native AnPrx6 protein harbours 212 amino acids (accession code: WP_010998539.1). The *ahpC* gene (accession code: *alr4404*) that codes for the AnPrx6 protein from *Anabaena* sp. PCC 7120, also known as *Nostoc* sp. PCC7120, was extracted from genomic DNA by PCR. The purified PCR fragment was digested with EcoRI and NotI and ligated into a pGEX-5X-2 GST expression vector (GE Healthcare) digested with the same enzymes^37^. *Escherichia coli* (*E. coli*) strains DH5α and BL21(DE3) were used as hosts for vector amplification and protein expression, respectively. The recombinant AnPrx6 protein was purified and crystallized as described by Mishra *et al*. (2011)^38^. Further details on expression, purification, crystallization of AnPrx6 are provided in the *Supporting Information (SI) Material and Methods*.

### X-ray data collection and processing

Crystals of AnPrx6 were vitrified in a nitrogen gas stream maintained at 100 K (Oxford CryoSystems Ltd, UK) and tested for diffraction at the in-house X-ray source (X8Proteum system, Bruker AXS). The best diffracting crystals were preserved in liquid nitrogen for later use. X-ray diffraction data sets were collected from cryo-cooled AnPrx6 crystals at a wavelength λ = 0.9793 Å onto an ADSC Q315R detector at beam-line ID23-1^67^ of the European Synchrotron Radiation Facility, Grenoble, France. The diffracted intensities were indexed and integrated with XDS^68^, prepared for scaling with pointless^69^. Aimless^70^ was used to scale and merge the intensities which were converted to structure factors with cTruncate^71^. About 5% of the data was marked for R_free_ calculation. (For crystallographic details and statistics see Supplementary Table 1).

### Structure determination and refinement

Phases were obtained by molecular replacement (MR) using the on-line version of Auto-Rickshaw^72^ (http://www.embl-hamburg.de/Auto-Rickshaw/) using monomer A of human Prx6 (hORF6, PDB code 1PRX^42^) as the search model. The CCP4 program suite^71^ was used for data analysis and manipulation. Iterative rounds of manual model building with Coot^73^ (version 0.8.2-pre rev. 5817) and refinement with phenix.refine^74,75^ of the Phenix program package (version 1.10.1–2155) were carried out until the R-free factor and R-factor converged. The data processing and refinement statistics are listed in the Supplementary Table 1. The atomic coordinates and structure factors for AnPrx6 have been deposited with the Worldwide Protein Data Bank (wwPDB) under code 5M6T. Additional ensemble refinement was carried out using the Ensemble refinement [alpha] program^76^ within the Phenix program package (version 1.10.1-2155).

### Sequence and structure analysis

Sequence searches against the non-redundant protein sequence database (nr) at NCBI^77^ and the Protein Data Bank (PDB)^41^ were performed with BLASTp^40^. Multiple sequence alignments of Prx proteins and subsequent phylogenetic tree analysis were carried with Clustal Omega^39^ at the European Bioinformatics Institute, EBI, (http://www.ebi.ac.uk/Tools/msa/clustalo/). For Prx subfamily classification the PREX database^9^ was consulted (http://csb.wfu.edu/prex.test/).

Structural analysis was carried out with Coot^73,78^ and ICM-Pro (MolSoft LLC)^79,80^. ICM-Pro was further used for multiple global structure superimpositions and to create the structure anchored multiple sequence alignment (saMSA), for which only structures with at least 30% sequence identity to AnPrx6 were considered. The PDB codes and further details about the superimposed structures are provided in the *SI Material and Methods* section.

All molecular graphic rendering was carried out with ICM-Pro (MolSoft LLC) or CCP4MG^78^.

### Molecular dynamics simulations

The simulation system was prepared based on the determined X-ray structure of AnPrx6 (PDB ID: 5M6T), in which the first dimer (i.e., chains A and B) was used. As  suggested by the electron density map the peroxidatic Cys (Cys45) was modelled as the triply oxidized cysteine-sulfonic acid in both monomers. Protonation states of all ionisable residues were assigned based on their pKa values or hydrogen bond interactions deduced from the X-ray structure. The positions of hydrogen atoms were determined using the HBUILD facility of the CHARMM program^81^ (version c38a2). All crystal waters were included and the resulting system was further solvated with a 99.0 Å rhombic dodecahedron (RHDO) box of TIP3P waters^82^ which gives at least 9 Å of solvation between the edge of the protein to the simulation box boundary along the longest protein axis. Then, 53 Na^+^ and 41 Cl^−^ ions were added at random positions to neutralize and to make the total ionic concentration of the final system to 0.15 M, resulting in a total of 67,003 atoms.

The resulting system was first energy minimized to avoid starting the molecular dynamics (MD) simulations with bad contacts between atoms in the system. A total of 5,000 steps of energy minimizations were performed with varying constraints and restraints: (1) 500 steps of minimization of all hydrogen atoms, (2) 1000 minimization steps with all protein heavy atoms fixed at the position of the X-ray structure, (3) 2000 steps of minimization with the harmonic position restraints applied to all protein heavy atoms, and (4) finally, 1500 steps of minimization without any restraints. The system was then heated slowly from 0 K to 300 K over 24 ps, followed by 150 ns production MD simulations. In all calculations, the all-atom CHARMM22 force fields^83^ with the CMAP correction^84^ applied to the protein backbone dihedrals were used to represent the protein and ions. The water molecules were represented by the TIP3P water model^82^. The force fields parameters for the oxidized Cys45 were developed based on the CHARMM General force fields^85^. All simulations were carried out using the RHDO periodic boundary conditions. Electrostatic interactions were evaluated using the smooth particle mesh Ewald (PME) summation method^86^ with 100 × 100 × 100 fast Fourier transform grid and real space interactions evaluated using the 11.0 Å cut-off. The same cut-off distance was used in the evaluation of van der Waals interactions, in which the interactions were switched off smoothly to zero between 9.0 Å and 11.0 Å. The MD simulations were performed with 2 fs integration time step, during which the temperature was maintained at 300 K using the Langevin thermostat (i.e., the NVT condition) with the 1 ps^−1^ friction coefficient, and the SHAKE-like algorithm^87^ applied to constrain bonds involving hydrogens. All MD simulations were performed using the NAMD program^88^ and the system preparation, energy minimizations, and trajectory analysis using the CHARMM program, respectively.

### Chaperone activity assay

The assay is based on the thermal aggregation of lysozyme and follows the protocol established by Ferreira *et al*.^60^. We used 360 μl of an aqueous solution containing 50 mM NaP*i* buffer pH 7.1 and 30 mM DTT and placed it for 5 minutes in a water bath maintained at 43 °C. Thereafter, a mixture of AnPrx6 and Hen Egg White Lysozyme were added to the solution at a final AnPrx6:Lysozyme molar ratio adjusted to 1:32 and a final reaction volume of 400 μl. After the addition of AnPrx6 and Lysozyme, the reaction temperature was maintained at 43 °C and the level of light scattered off the reaction mixture was measured every 30 seconds over a period of 20 minutes at a wavelength of 360 nm using a micro-plate spectrophotometer (SPECTRA max, 190 USA). Negative control experiments, determining the baseline for scattering, were performed using 400 μl of the buffer solution including DTT but excluding AnPrx6 or Lysozyme. Positive control experiments were performed by replacing AnPrx6 with BSA.

### Data Availability

The atomic coordinates and structure factors for AnPrx6 from *Anabaena* sp. PCC 7120 have been deposited with the Worldwide Protein Data Bank (wwPDB) under code 5M6T.

## Electronic supplementary material

Supplementary Information

Supplementary simulation Sim1

Supplementary simulation Sim3
