# Author Correction: Active-site plasticity revealed in the asymmetric dimer of AnPrx6 the 1-Cys peroxiredoxin and molecular chaperone from *Anabaena* sp. PCC 7120

**DOI:** 10.1038/s41598-018-26715-8

**Published:** 2018-05-30

**Authors:** Yogesh Mishra, Michael Hall, Roland Locmelis, Kwangho Nam, Christopher A. G. Söderberg, Patrik Storm, Neha Chaurasia, Lal Chand Rai, Stefan Jansson, Wolfgang P. Schröder, Uwe H. Sauer

**Affiliations:** 1 Department of Chemistry, Umeå University, SE-901 87 Umeå, Sweden; 2 Umeå Plant Science Centre, Department of Plant Physiology, Umeå University, SE-901 87 Umeå, Sweden; 3 Computational Life-Science Cluster, CLiC, Umeå University, SE-901 87 Umeå, Sweden; 4 Department of Chemistry and Biochemistry, University of Texas at Arlington, Arlington, TX 76019-0065 USA; 5 MAX IV Laboratory, CoSAXS beamline, Lund University, Fotongatan 2, SE-225 94 Lund, Sweden; 6 Molecular Biology Section, Laboratory of Algal Biology, Centre of Advanced Study in Botany, Institute of Science, Banaras Hindu University, Varanasi, 221005 India; 7 Department of Biotechnology and Bioinformatics, North Eastern Hill University, Shillong, 793022 India; 8 Present Address: Department of Botany, Centre of Advanced Study in Botany, Institute of Science, Banaras Hindu University, Varanasi, 221005 India

Correction to: *Scientific Reports* 10.1038/s41598-017-17044-3, published online 07 December 2017

The original version of this Article contained an error in the title of the paper, where “*Anabaena* sp. PCC 7120” was incorrectly given as “*Anabaena* sp. PCC 7210”. This has now been corrected in the PDF and HTML versions of the Article, and in the accompanying Supplementary Information file.

