# Supplementary material for: Active-site plasticity revealed in the asymmetric dimer of AnPrx6 the 1-Cys peroxiredoxin and molecular chaperone from Anabaena sp. PCC 7120
Source: Sci Rep. 2017 Dec 7;7:17151. doi: 10.1038/s41598-017-17044-3 (PMC5719442; doi:10.1038/s41598-017-17044-3)
Supplement: Supplementary file 1 — Supplementary Information [file 41598_2017_17044_MOESM1_ESM.pdf]

Supplementary information for:
Active-site plasticity revealed in the asymmetric dimer of AnPrx6 the 1-Cys peroxiredoxin and molecular chaperone from *Anabaena* sp. PCC 7120

Yogesh Mishra[1,2,3], Michael Hall[1], Roland Locmelis[1], Kwangho Nam[1,4,5], Christopher A. G. Söderberg[6], Patrik Storm[1], Neha Chaurasia[7,8], Lal Chand Rai[7], Stefan Jansson[2], Wolfgang P. Schröder[1,2] and Uwe H. Sauer[1,4*]

Affiliations:
1. Department of Chemistry, Umeå University, SE-901 87 Umeå, Sweden
2. Umeå Plant Science Centre, Department of Plant Physiology, Umeå University, SE-901 87 Umeå, Sweden
3. Current address: Department of Botany, Centre of Advanced Study in Botany, Institute of Science, Banaras Hindu University, Varanasi, India-221005.
4. Computational Life-Science Cluster, CLiC, Umeå University, SE-901 87 Umeå, Sweden
5. Department of Chemistry and Biochemistry University of Texas at Arlington, Arlington, TX 76019-0065 USA
6. MAX IV Laboratory, CoSAXS beamline, Lund University, Fotongatan 2, SE-225 94 Lund, Sweden
7. Molecular Biology Section, Laboratory of Algal Biology, Centre of Advanced Study in Botany, Institute of Science, Banaras Hindu University, Varanasi-221005, India
8. Department of Biotechnology and Bioinformatics, North Eastern Hill University Shillong-793022, India
* To whom correspondence should be addressed: uwe.sauer@chem.umu.se

Supplementary Materials and Methods

Cloning, expression, purification and crystallization of AnPrx6. The cloning of the *ahpC* gene (*alr4404*) from *Anabaena* sp. PCC7120, also called *Nostoc* sp. PCC7120, was carried out as described by Mishra et al. (2009)[1]. The AnPrx6 protein harbours 212 amino acids (accession code: WP_010998539.1). Recombinant AnPrx6 was expressed in *E. coli*, BL21(DE3) (Novagen), purified and crystallized as described previously[2]. Briefly, the *ahpC* gene was extracted from genomic DNA by PCR. The purified PCR fragment was digested with EcoRI and NotI and ligated into a pGEX-5X-2 GST expression vector (GE Healthcare) digested with the same enzymes. The vector was amplified in *Escherichia coli* (*E. coli*) strain DH5α. The *E. coli* strain BL21(DE3) was used for the expression of recombinant GST-AnPrx6 fusion protein, which was purified by affinity chromatography. The GST-fusion tag was cleaved off with 10 U of Factor-Xa (GE Healthcare) per mg of AnPrx6. As an artefact from the cloning and protein cleavage procedure, the final recombinant AnPrx6 protein contains six residual non-native amino acids (GIPGIP) preceding the starting methionine. Thus, the purified protein has a calculated molecular weight of 24.23 kDa, a theoretical extinction coefficient of 33920 (M^{-1} cm^{-1}) and a predicted isoelectric point (pI) value of 5.1, as determined with the ExPASy Protparam tool[3]. Following size exclusion chromatography using a Sephacryl S-300-HR column equilibrated in a buffer containing 150 mM NaCl, 1 mM $CaCl_2$ and 50 mM Tris-HCl at pH 7.5, the protein was concentrated to 13.0 mg/ml, as determined by the Bradford assay using Bovine Serum Albumin, BSA, as the standard (Sigma, USA). The purified protein was then either used directly for crystallization set-ups or shock-cooled into liquid nitrogen and stored at -80°C. Single diffraction-quality crystals were

grown by the vapour diffusion hanging drop method at 291 K[2]. The crystallization drops were made by mixing 2 µl AnPrx6 protein at 13 mg/ml with 2 µl crystallization buffer, (0.2 M sodium acetate trihydrate, 0.1 M sodium cacodylate trihydrate pH 6.5 and 20% (w/v) polyethylene glycol 8000). The drops were equilibrated against 500 µl of the same buffer. Crystals grew in about one month to a size of approximately 0.6 • 0.1 • 0.03 mm^3.

Structure anchored sequence alignment and structure analysis. ICM-Pro (MolSoft LLC)[4,5] was used for multiple global structure superimpositions and to create the structure anchored multiple sequence alignment (saMSA), for which only structures with at least 30% sequence identity to AnPrx6 were considered.
In the following, the number triplets in parenthesis next to the PDB code stand for: the pairwise sequence identity (%) / the number of aligned residues / the root mean square deviation (Å) after superimposing chain A of the respective PDB file onto chain A of AnPrx6. The following PDB entries were superimposed onto AnPrx6: *Homo sapiens* Prx6 (hORF6)[6], 1PRX (52/200/1.1); *Arenicola marina* Prx6[7], 2V2G (48/199/1.3); *Plasmodium yoelii* Prx6[8], 1XCC (41/198/1.4); *Aeropyrum pernix* K1 Prx6[9], 2CV4 (39/193/1.7); *Pyrococcus horikoshii* Prx6[10], 3W6G (38/187/1.6); *Trypanosoma cruzi* tryparedoxin peroxidase (Prx1)[11], 4LLR (34/165/1.2); *Rattus norwegicus* heme-binding protein 23 kDa (Prx1)[12], 1QQ2 (30/148/1.6); *Leishmania major* tryparedoxin peroxidase I (Prx1)[13], 3TUE (32/144/1.5); *Crithidia fasciculate* tryparedoxin peroxidase (Prx1)[14], 1E2Y (31/146/1.4); *Homo sapiens* thioredoxin peroxidase-B (Prx1)[15], 1QMV (30/166/1.4); *Schistosoma mansoni* peroxiredoxin I (Prx1)[16], 3ZTL (36/162/1.5); *Ancylostoma ceylanicum* peroxiredoxin-1 (Prx1)[17], 4FH8 (33/148/1.7); *Helicobacter pylori* alkyl hydroperoxide-reductase (AhpC) (Prx1)[18], 1ZOF (32/147/2.0). All molecular graphic rendering was carried out with ICM-Pro (MolSoft LLC) or CCP4MG[19].

Size-exclusion chromatography. The oligomeric state of AnPrx6 at room temperature was determined by analytical size exclusion chromatography (SEC) using a high resolution Superdex 200 10/300 column (GE Healthcare, Sweden). Prior to each run, the column was equilibrated with the buffer used to store the AnPrx6 protein, containing 50 mM Tris-HCl pH 7.5, 150 mM NaCl, and 1 mM $CaCl_2$. For each run, a sample loop with a capacity of 100 µl was filled with AnPrx6 protein at a concentration of either 0.1 mg ml^{-1}, 1.0 mg ml^{-1} or 10.0 mg ml^{-1}, and loaded onto the column, resulting in 0.01 mg, 0.1 mg or 1.0 mg of protein passing through the column. Retention curves at a wavelength of 280 nm were recorded in the absence or presence of 20 mM H_2O_2 in the buffer. A standard curve for the Superdex 200 10/300 column using the above buffer condition was generated from the elution profile of a standard protein mix (BioRad Gel Filtration Standard, catalog # 151-1901). The standard curve was used to estimate the molecular weight of the eluted AnPrx6 protein.

Small Angle X-ray Scattering (SAXS) data collection: SAXS measurements were carried out in a buffer containing 50 mM TRIS-HCl pH 8.0, 150 mM NaCl, 1 mM $CaCl_2$, without DTT, at two AnPrx6 concentrations (1.6 mg/ml and 4.8 mg/ml). All samples were centrifuged prior to measurements. The SAXS data sets of AnPrx6 were collected at the MAX IV Laboratory, MAX II beamline I911-SAXS[20]. The intensity data was recorded on a hybrid pixel Pilatus 1M detector as a function of the scattering vector **q** ($q = (4\pi \sin\theta)/\lambda$). Data reduction, normalization to the intensity of the transmitted beam, and buffer subtraction were performed using beamline tools and scripts. The Primus program in the ATSAS software package[21,22] was used to estimate forward scattering I(0), radius of gyration, pair-distance distribution function, and the excluded volume of the hydrated particle (Porod volume)[21,23]. Molecular weight estimations were made using Lysozyme as a molecular weight standard, as well as the Porod volume.

Peroxidase activity assay. Peroxidase activity of AnPrx6 was determined using the ammonium ferrous sulphate and xylenol orange (FOX) assay[24]. The assay was performed in triplicates at 23°C in 100 µl reaction buffer containing 20 mM NaP*i* pH 7.0, 150 mM NaCl, and 1 mM DTT and a protein concentration of 4 µM. The reaction was initiated by adding hydrogen peroxide (H_2O_2) to a final concentration of 600 µM. After exactly one minute, 5 µl of the reaction mixture was removed and added to 500 µl of the FOX reagent solution, consisting of 250 µM ammonium ferrous sulphate, 100 µM xylenol orange, 25 mM H_2SO_4 and 100 mM sorbitol, which terminated the reaction. After incubation for one hour in the dark, 500 µl of deionized water was added to the FOX assay mixture and the absorbance of the final solution was measured at a wavelength of 560 nm. The enzymatic consumption of hydrogen peroxide due to the AnPrx6 protein in the sample was determined with the help of a standard curve that shows the absorption at 560 nm plotted as a function of increasing H_2O_2 concentrations. Control experiments were performed using samples containing no AnPrx6 in order to account for the potential background signal originating from the constituents of the reaction buffer (Supplementary Fig. S8).

To determine the rate of H_2O_2 consumption with respect to the enzyme concentration, FOX assays in the same buffer as before were performed at AnPrx6 concentrations increasing from 0 µM to 60 µM and a fixed, saturating H_2O_2 concentration of 600 µM (Supplementary Fig. S9).

Supplementary Figures and Tables

Supplementary Table S1. Data collection, processing and refinement statistics

	AnPrx6
**Data collection**	
Wavelength [Å]	0.97929
Space group (Nr.)	$P2_12_12_1$ (19)
Unit cell dimensions	
a, b, c [Å]	80.09, 102.07, 109.74
α, β, γ [°]	90, 90, 90
Molecules per asymmetric unit	4
Resolution range (highest shell) [Å]	51.0 – 2.3 (2.38 – 2.30)
Accepted reflections	151063 (7398)
Unique reflections	39398 (3204)
Completeness [%]	97 (82)
Multiplicity	3.8 (2.3)
Mean ($I/\sigma I$)	12.6 (2.2)
R_{merge} [%]	8.3 (43.1)
R_{meas} [%]	9.5 (55.0)
R_{pim} [%]	4.7 (33.3)
$CC_{1/2}$ [%]	99.6 (81.3)
CC* [%]	99.9 (94.7)
**Refinement**	
Resolution range (highest shell) [Å]	51.05 - 2.30 (2.36 – 2.30)
Reflections used for refinement	39327 (3204)
Reflections used for R_{free}	1970 (143)
R_{work} [%]	20.4/ (27.4)
R_{free} [%]	24.5 / (33.3)
Overall map CC (Fc, 2mFo-DFc) [%]	87.5
R. m. s. deviations	
Bond length [Å]	0.002
Bond angles (°)	0.452
Ramanchandran plot statistics	
Residues in favored region [%]	95
Residues in allowed region [%]	4.5
Residues in outlier region [%]	0.4
All-atom clash score	0.9
No. of non-hydrogen atoms	7078
Protein (non-hydrogen)	6738
Solvent (H_2O) (non-hydrogen)	340
Average B-factors (Å^2):	31.2
Protein chains	31.2
Water molecules	30.8

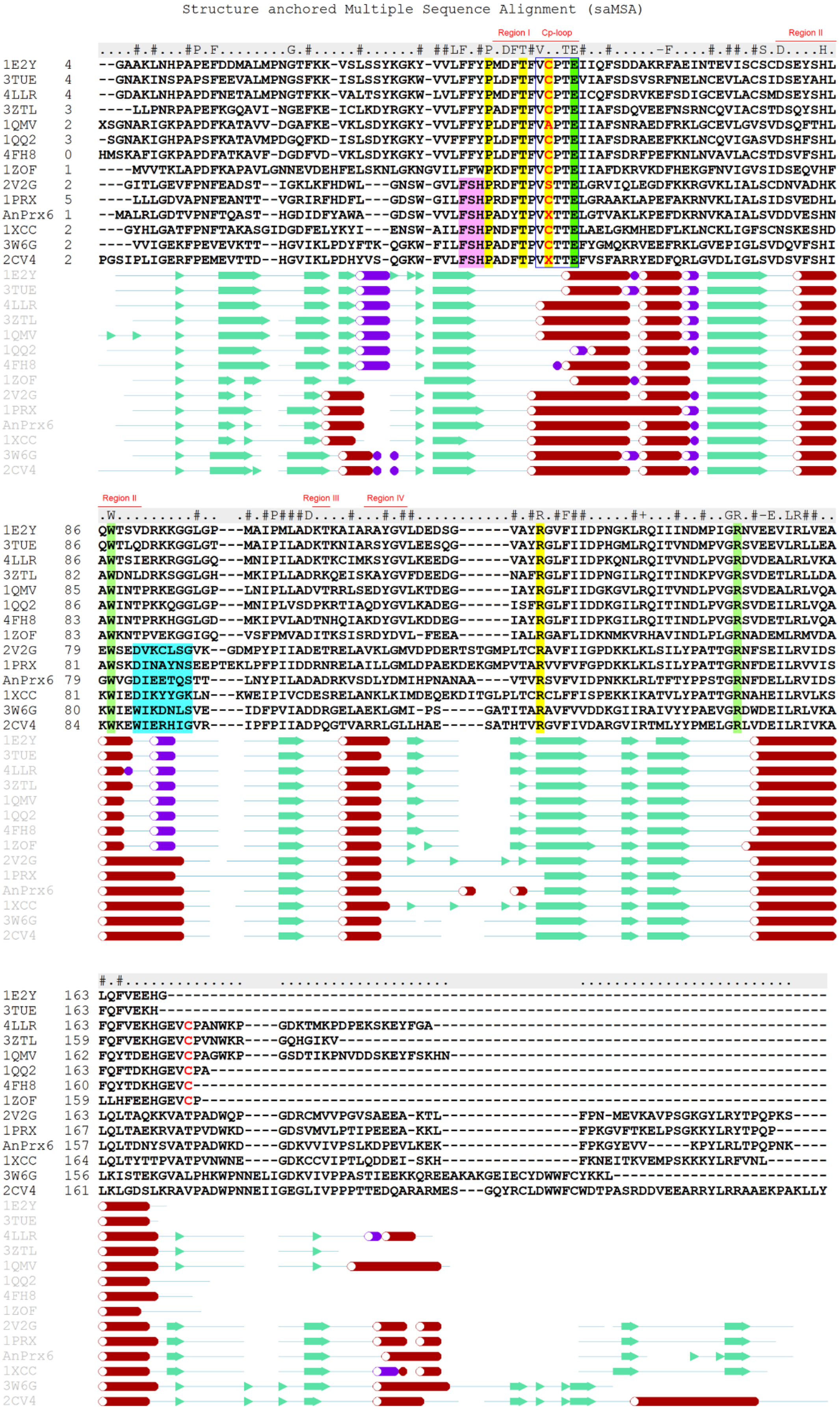
Structure anchored Multiple Sequence Alignment (saMSA)
Region I
Cp-loop
Region II
....#.#...#P.F........G.#.....#......# ##LF.#P.DFT#V..TE#..#.....-F....#.##.#S.D....H.
1E2Y 4 --GAAKLNHPAPEFDDMALMPNGTFKK-VSLSSYKGKY-VVLFFYPMDFTFVCPTEIIQFSDDAKRFAEINTEVISCSCDSEYSHL
3TUE 4 --GNAKINSPAPSFEEVALMPNGSFKK-ISLSSYKGKW-VVLFFYPLDFTFVCPTEVIAFSDSVSRFNELNCEVLACSIDSEYAHL
4LLR 4 --GDAKLNHPAPDFNETALMPNGTFKK-VALTSYKGKW-LVLFFYPMDFTFVCPTEICQFSDRVKEFSDIGCEVLACSMDSEYSHL
3ZTL 3 ----LLPNRPAPEFKGQAVI-NGEFKE-ICLKDYRGKY-VVLFFYPADFTFVCPTEIIAFSDQVEEFNSRNCQVIACSTDSQYSHL
1QMV 2 XSGNARIGKPAPDFKATAVV-DGAFKE-VKLSDYKGKY-VVLFFYPLDFTFVAPTEIIAFSNRAEDFRKLGCEVLGVSVDSQFTHL
1QQ2 3 -SGNAKIGHPAPSFKATAVMPDGQFKD-ISLSDYKGKY-VVFFFYPLDFTFVCPTEIIAFSDRAEEFKKLNCQVIGASVDSHFSHL
4FH8 0 HMSKAFIGKPAPDFATKAVF-DGDFVD-VKLSDYKGKY-VVLFFYPLDFTFVCPTEIIAFSDRFPEFKNLNVAVLACSTDSVFSHL
1ZOF 1 ----MVVTKLAPDFKAPAVLGNNEVDEHFELSKNLGKNGVILFFWPKDFTFVCPTEIIAFDKRVKDFHEKGFNVIGVSIDSEQVHF
2V2G 2 ---GITLGEVFPNFEADST--IGKLKFHDWL---GNSW-GVLFSHPRDFTPVSTTELGRVIQLEGDFKKRGVKLIALSCDNVADHK
1PRX 5 ----LLLGDVAPNFEANTT--VGRIRFHDFL---GDSW-GILFSHPRDFTPVCTTELGRAAKLAPEFAKRNVKLIALSIDSVEDHL
AnPrx6 1 --MALRLGDTVPNFTQAST--HGDIDFYAWA---GDSW-VVLFSHPADYTPVXTTELGTVAKLKPEFDKRNVKAIALSVDDVESHN
1XCC 2 ---GYHLGATFPNFTAKASGIDGDFELYKYI---ENSW-AILFSHPNDFTPVCTTELAELGKMHEDFLKLNCKLIGFSCNSKESHD
3W6G 2 ----VVIGEKFPEVEVKTT--HGVIKLPDYFTK-QGKW-FILFSHPADFTPVCTTEFYGMQKRVEEFRKLGVEPIGLSVDQVFSHI
2CV4 2 PGSIPLIGERFPEMEVTTD--HGVIKLPDHYVS-QGKW-FVLFSHPADFTPVXTTEFVSFARRYEDFQRLGVDLIGLSVDSVFSHI
1E2Y
3TUE
4LLR
3ZTL
1QMV
1QQ2
4FH8
1ZOF
2V2G
1PRX
AnPrx6
1XCC
3W6G
2CV4
Region II
Region III
Region IV
.W.........#.. .#.#P###D....#...#.##...........#.#R.#F##.....#+...#..#..GR.#-E.LR##..
1E2Y 86 QWTSVDRKKGGLGP---MAIPMLADKTKAIARAYGVLDEDSG------VAYRGVFIIDPNGKLRQIIINDMPIGRNVEEVIRLVEA
3TUE 86 QWTLQDRKKGGLGT---MAIPILADKTKNIARSYGVLEESQG------VAYRGLFIIDPHGMLRQITVNDMPVGRSVEEVLRLLEA
4LLR 86 AWTSIERKRGGLGQ---MNIPILADKTKCIMKSYGVLKEEDG------VAYRGLFIIDPKQNLRQITVNDLPVGRDVDEALRLVKA
3ZTL 82 AWDNLDRKSGGLGH---MKIPLLADRKQEISKAYGVFDEEDG------NAFRGLFIIDPNGILRQITINDKPVGRSVDETLRLLDA
1QMV 85 AWINTPRKEGGLGP---LNIPLLADVTRRLSEDYGVLKTDEG------IAYRGLFIIDGKGVLRQITVNDLPVGRSVDEALRLVQA
1QQ2 86 AWINTPKKQGGLGP---MNIPLVSDPKRTIAQDYGVLKADEG------ISFRGLFIIDDKGILRQITINDLPVGRSVDEILRLVQA
4FH8 83 AWINTPRKHGGLGD---MKIPVLADTNHQIAKDYGVLKDDEG------IAYRGLFIIDPKGILRQITINDLPVGRSVDETLRLVQA
1ZOF 83 AWKNTPVEKGGIGQ---VSFPMVADITKSISRDYDVL-FEEA------IALRGAFLIDKNMKVRHAVINDLPLGRNADEMLRMVDA
2V2G 79 EWSEDVKCLSGVK--GDMPYPIIADETRELAVKLGMVDPDERTSTGMPLTCRAVFIIGPDKKLKLSILYPATTGRNFSEILRVIDS
1PRX 81 AWSKDINAYNSEEPTEKLPFPIIDDRNRELAILLGMLDPAEKDEKGMPVTARVVFVFGPDKKLKLSILYPATTGRNFDEILRVVIS
AnPrx6 79 GWVGDIEETQSTT----LNYPILADADRKVSDLYDMIHPNANAA----VTVRSVFVIDPNKKLRLTFTYPPSTGRNFDELLRVIDS
1XCC 81 KWIEDIKYYGKLN---KWEIPIVCDESRELANKLKIMDEQEKDITGLPLTCRCLFFISPEKKIKATVLYPATTGRNAHEILRVLKS
3W6G 80 KWIEWIKDNLSVE----IDFPVIADDRGELAEKLGMI-PS-----GATITARAVFVVDDKGIIRAIVYYPAEVGRDWDEILRLVKA
2CV4 84 KWKEWIERHIGVR----IPFPIIADPQGTVARRLGLLHAE-----SATHTVRGVFIVDARGVIRTMLYYPMELGRLVDEILRIVKA
1E2Y
3TUE
4LLR
3ZTL
1QMV
1QQ2
4FH8
1ZOF
2V2G
1PRX
AnPrx6
1XCC
3W6G
2CV4
#.#.............. ....
1E2Y 163 LQFVEEHG--
3TUE 163 FQFVEKH---
4LLR 163 FQFVEKHGEVCPANWKP---GDKTMKPDPEKSKEYFGA--
3ZTL 159 FQFVEKHGEVCPVNWKR---GQHGIKV---
1QMV 162 FQYTDEHGEVCPAGWKP---GSDTIKPNVDDSKEYFSKHN--
1QQ2 163 FQFTDKHGEVCPA---
4FH8 160 FQYTDKHGEVC---
1ZOF 159 LLHFEEHGEVCP--
2V2G 163 LQLTAQKKVATPADWQP---GDRCMVVPGVSAEEA-KTL----------------FPN-MEVKAVPSGKGYLRYTPQPKS----
1PRX 167 LQLTAEKRVATPVDWKD---GDSVMVLPTIPEEEA-KKL----------------FPKGVFTKELPSGKKYLRYTPQP------
AnPrx6 157 LQLTDNYSVATPADWKD---GDKVVIVPSLKDPEVLKEK----------------FPKGYEVV-----KPYLRLTPQPNK----
1XCC 164 LQLTYTTPVATPVNWNE---GDKCCVIPTLQDDEI-SKH----------------FKNEITKVEMPSKKKYLRFVNL-------
3W6G 156 LKISTEKGVALPHKWPNNELIGDKVIVPPASTIEEKKQREEAKAKGEIECYDWWFCYKKL-------------------------
2CV4 161 LKLGDSLKRAVPADWPNNEIIGEGLIVPPPTTEDQARARMES---GQYRCLDWWFCWDTPASRDDVEEARRYLRRAAEKPAKLLY
1E2Y
3TUE
4LLR
3ZTL
1QMV
1QQ2
4FH8
1ZOF
2V2G
1PRX
AnPrx6
1XCC
3W6G
2CV4

Figure S1. Structure anchored multiple sequence alignment (saMSA). AnPrx6 and thirteen Prx proteins with known crystal structures and at least 30% sequence identity, compared to AnPrx6, were simultaneously superimposed. The respective PDB codes precede each sequence, except for AnPrx6. Identical amino acids are indicated by their single letter code above the alignment. Similar amino acids are marked with a hash (#). Highlighted in yellow are the conserved residues of the catalytic PxxxTxxC motif[25,26], and the fully conserved active site arginine. In AnPrx6 these are residues Pro38, Thr42, Cys45 and Arg122. The peroxidatic cysteine (C_P) is shown in red and the blue frame marks the residues of the active site C_P-loop. C_P was mutated to Ala and Ser in 1QMV and 2V2G, respectively and was oxidized to Cysteine sulfonic acid (X) in AnPrx6 and 2CV4. If present, the distal resolving Cys (C_R) is marked red. Additional amino acids that contribute to the active site environment are Phe35, His37, Pro43, Val44, Glu48, Leu49, Trp80, Pro141 and Arg145. Of these, Glu48, Trp80, and Arg145 are fully conserved and highlighted in green. Highly conserved among the Prx6 proteins is the FSH motif, highlighted in pink, just before PxxxTxxC motif. The four regions (I-IV) marked with red above the alignment indicate those residues of 2-Cys Prxs that are involved in decamer formation[27]. Highlighted in light blue are seven residues of Prx6 proteins that extend helix α3 by almost two turns compared to other Prxs, which causes the loop between α3 and β5 to adopt a conformation that interferes with decameric ring formation. The succession of secondary structure elements for each protein is indicated below the saMSA: aquamarine arrows depict β-strands, red tubes represent α-helices and magenta tubes 3_{10} helical turns.

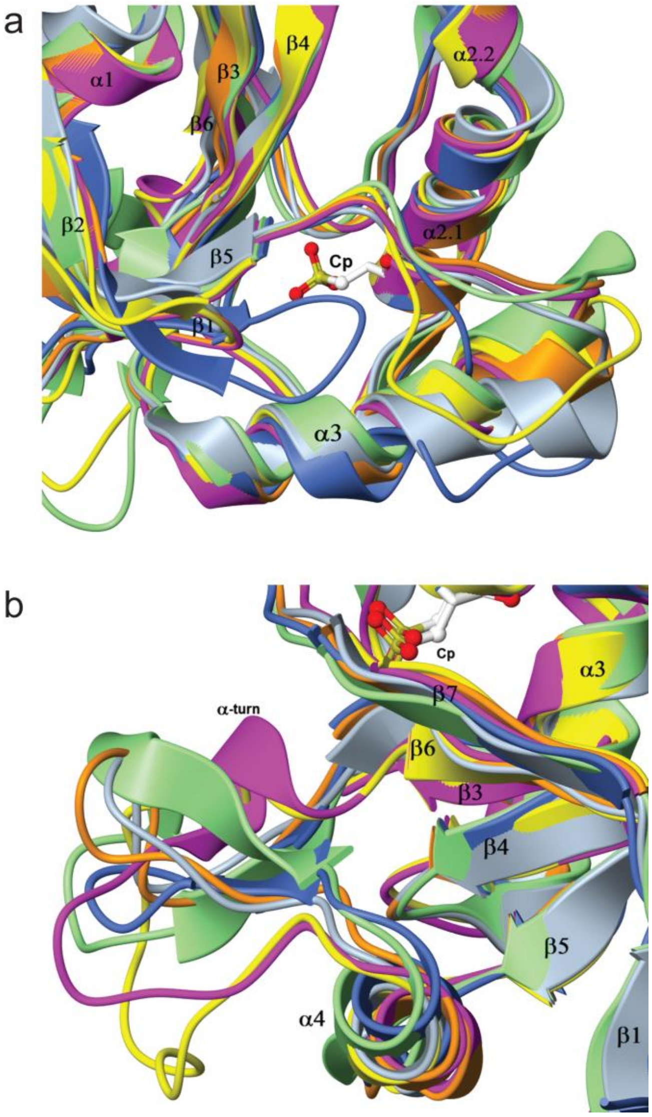

Figure S2. Ribbon diagrams showing structural variation. Superimposed are the backbone atoms of the AnPrx6 monomer A (purple), AnPrx6 monomer B (orange), human ORF6 (1PRX, yellow), *Arenicola marina* Prx6 (2V2G, light green), *Aeropyrum pernix* Prx6 (2CV4, gray) and *Trypanozoma cruzi* (4LLR, blue). (**a**) The loop connecting helix α3 with strand β5. The length of helix α3 is similar in all Prx6 proteins, however the orientation of the helix axis can vary by as much as 25°, resulting in a repositioning of the C-terminal part of helix α3. The α3-β5 loops of AnPrx6 monomers A and B are similar, while they deviate from the corresponding loops in human Prx6 (1PRX), *Arenicola marina* Prx6 (2V2G) and *Aeropyrum pernix* Prx6 (2CV4). As a representative of 2-Cys Prxs that form $(\alpha_2)_5$ rings, the α3-β5 region from *Trypanozoma cruzi* (4LLR) is shown in blue. Typical for the $(\alpha_2)_5$ ring forming Prxs is that their helix α3 is about two turns shorter than the helix α3 in Prx6 and that the α3-β5 loop can form a 3_{10} helical turn. **(b)** The loop connecting α4 with β6. The loop spanning residues Asp109 to Arg122 of AnPrx6 contains an α-helical turn in monomers A and C (purple in Fig. 1a and 1b), while the same loop in monomer B and D (orange in Fig. 1b) adopts an extended, irregular shape which resembles the corresponding loop of *Aeropyrum pernix* Prx6 (2CV4). Other Prx proteins form a short two stranded β-sheet as can be seen in PDB entries 2V2G and 4LLR.

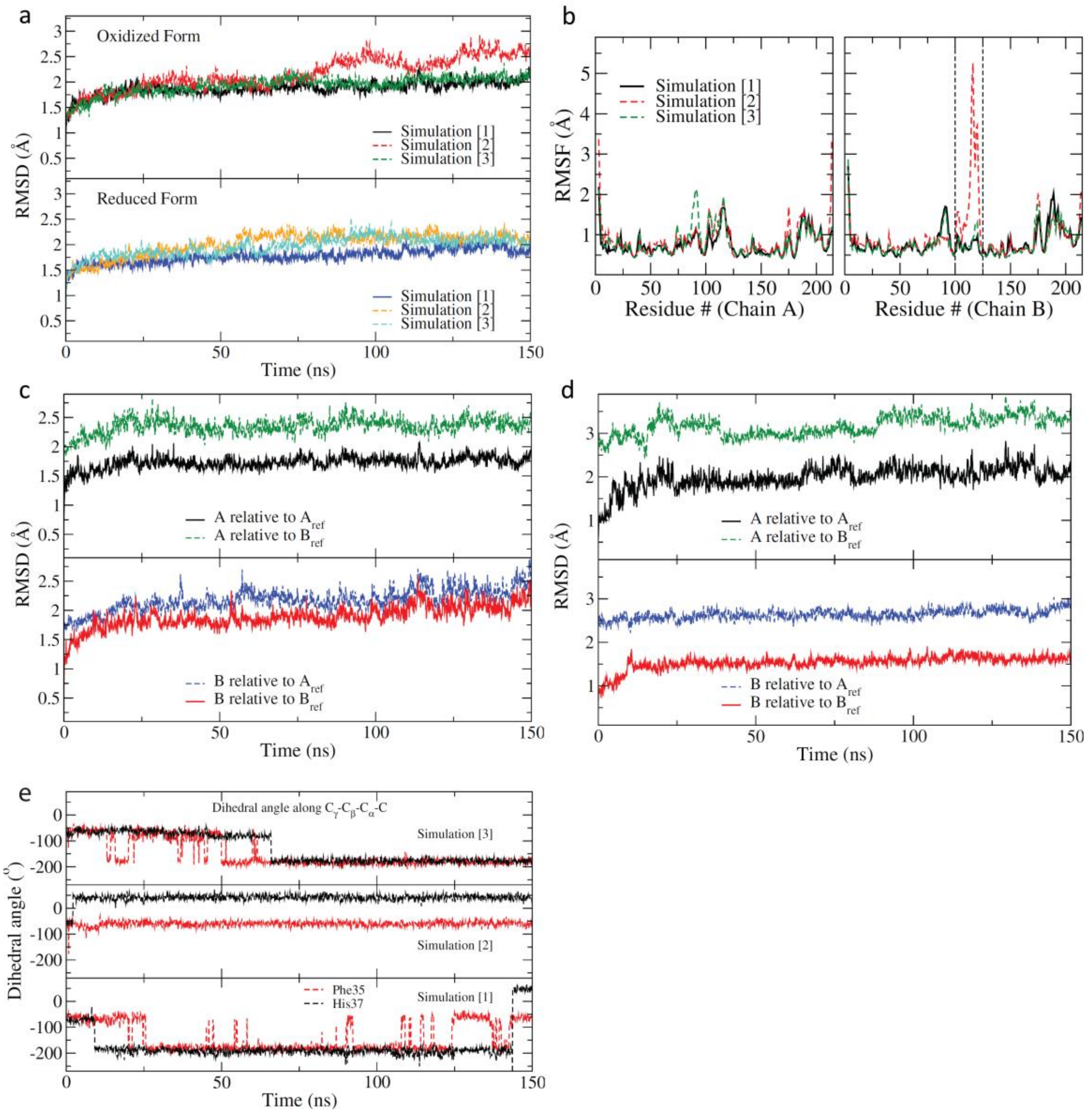

Figure S3. Analysis of MD simulation trajectories. (**a**) Root-mean-square deviation (RMSD) of atomic backbone positions, relative to the X-ray structure, calculated for three independent simulations (triplicates) spanning 150 ns. The top panel shows the RMSD for the oxidized active site Cys45 (Cys45-O_2-OH) and the bottom panel the RMSD for the fully reduced Cys45. (**b**) The C_α-carbon root-mean-square-fluctuation (RMSF) for the three fully oxidized Cys45 system simulations. For the chain B, the region showing the large RMSF in the Simulation [2] (from helix α4 to the beginning of β6) is highlighted by the vertical dashed lines. (**c**) RMSD of backbone atoms of monomers A and B relative to monomers A and B of the X-ray structure. A_{ref} and B_{ref} refer to the X-ray structures of chain A and B, respectively. (**d**) RMSD of the active site residues (residues 36 to 48, 107 to 127, and 137 to 149) relative to their corresponding X-ray structure positions of monomer A and B. (**e**) The side chain dihedral angles along C_γ-C_β-C_α-C of His37 and Phe35 in monomer B shown for the three oxidized Cys45 (Cys45-O_2-OH) simulations.

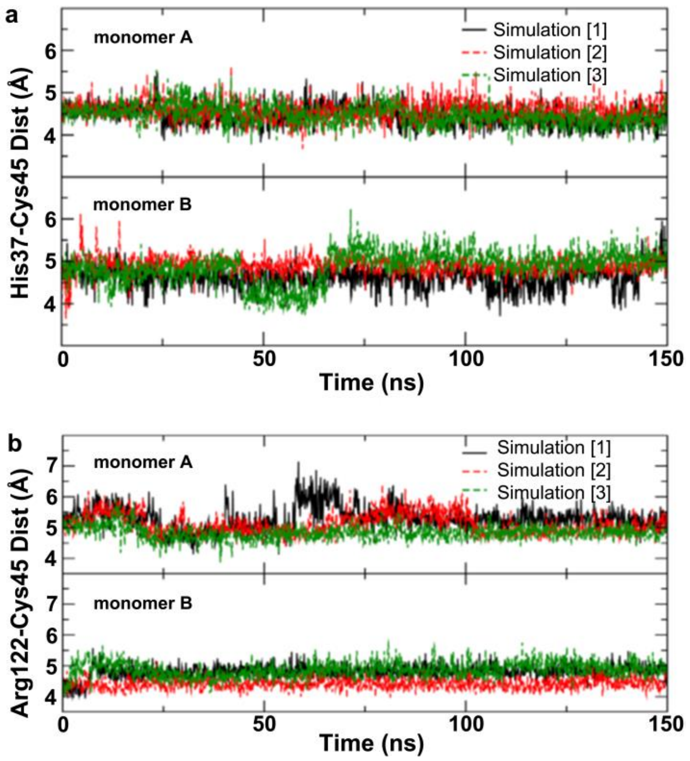

Figure S4. H-bond distance variations during MD simulations. (**a**) For the H-bond interaction between His37-Cys45, the His37 Cγ --- Cys45 Sγ H-bond distance was monitored. In general, the distance in monomer A (upper panel) does not fluctuate as much as in monomer B (lower panel), which shows slightly larger distance fluctuations and longer His37-Cys45 H-bond distances. (**b**) For the H-bond interaction between Arg122-Cys45, the Arg122 Cζ --- Cys45 Sγ H-bond distance was used. In this case monomer A (upper panel) shows larger distance fluctuations and longer Arg122-Cys45 H-bond distances compared to those of monomer B (lower panel).

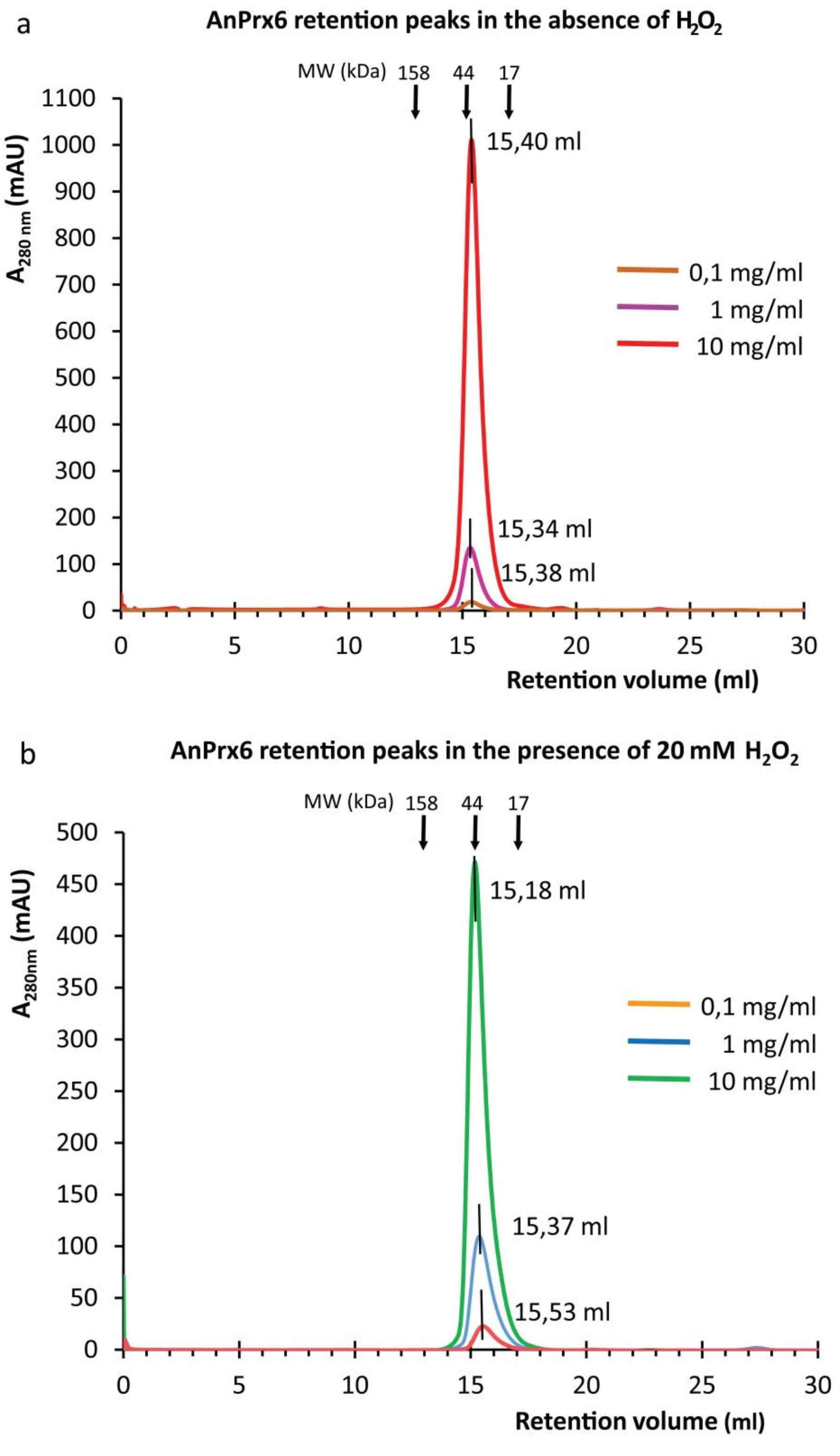

Figure S5. Size exclusion chromatography (SEC) of recombinant AnPrx6. (**a**) SEC analysis of AnPrx6 at protein concentrations of 0.1 mg ml^{-1}, 1.0 mg ml^{-1} and 10 mg ml^{-1} in the absence of H_2O_2 and (**b**) in the presence of 20 mM H_2O_2. In all cases, AnPrx6 elutes in a single peak with a derived average molecular weight of about 44,2 kDa without H_2O_2 (S5a) and 44,7 kDa with 20 ml H_2O_2 (S5b). These values correspond well with the calculated molecular weight for dimeric AnPrx6 (48.4 kDa). No other peaks were detected.

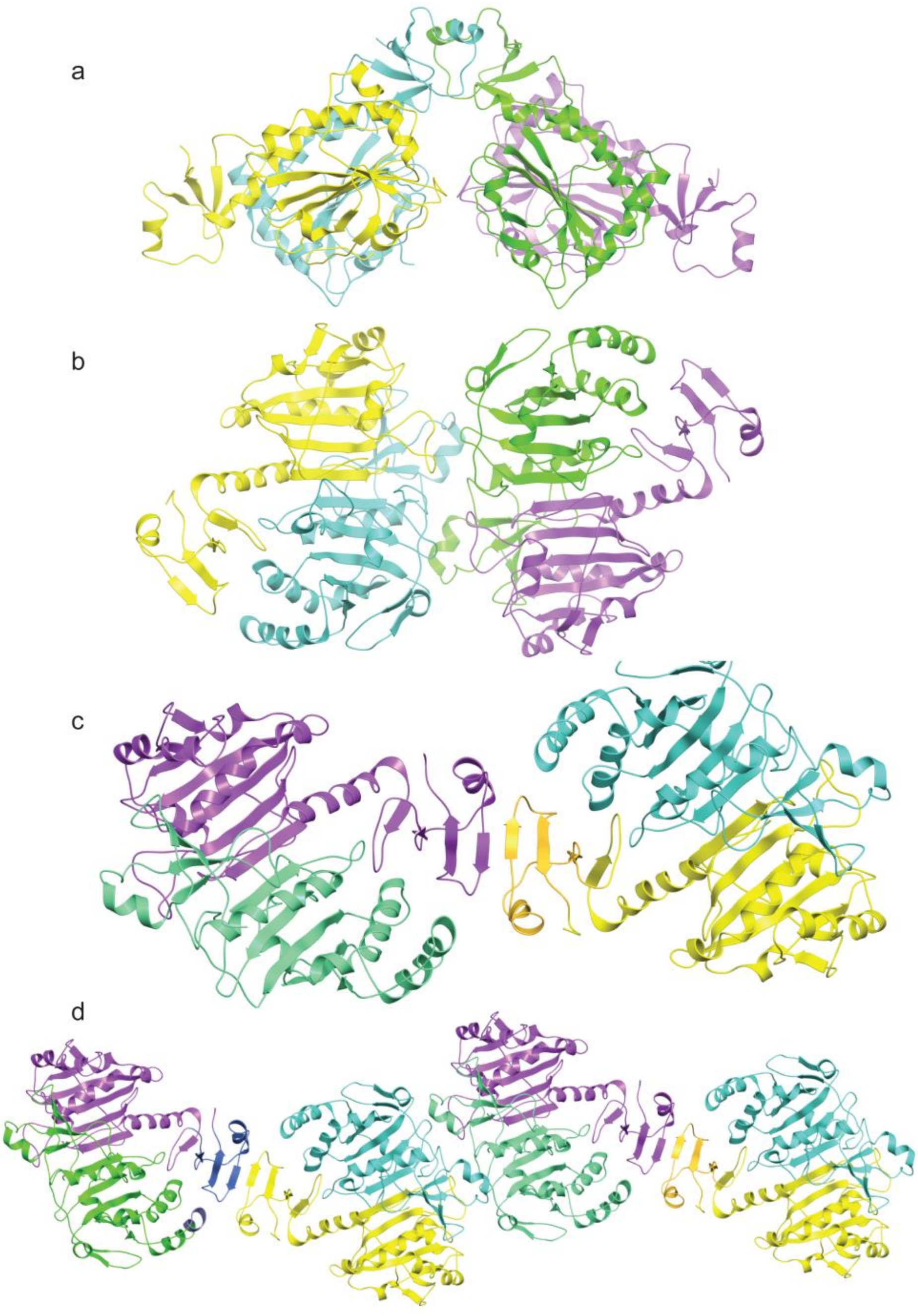

Figure S6. The asymmetric unit (a.s.u) of the AnPrx6 crystals in space-group $P2_12_12_1$ contains a dimer-of-dimers. The buried interface surface is relatively small (690 $Å^2$ as determined by PDBePISA) and is formed by residues from helix α6 of the C-terminal domain and to a lesser extent by residues from the N-terminal loop and the loop connecting helix α4 and strand β6. The dimer-of-dimers is likely due to crystal packing effects and is not biologically relevant. (**a**) Front view of the two dimers in the a.s.u. The left-hand dimer is coloured yellow and blue and the dimer on the right green and purple. (**b**) The dimer-dimer interaction viewed by rotating the molecules 90° around the horizontal axis passing through the centre of the two dimers. (**c**) Contacts between dimers from adjacent asymmetric units are in both case mediated through residues from strand β10 of the C-terminal domain, thus forming a continuous eight-stranded β-sheet. (**d**) Packing diagram showing the extended crystal packing interactions within one crystal plane.

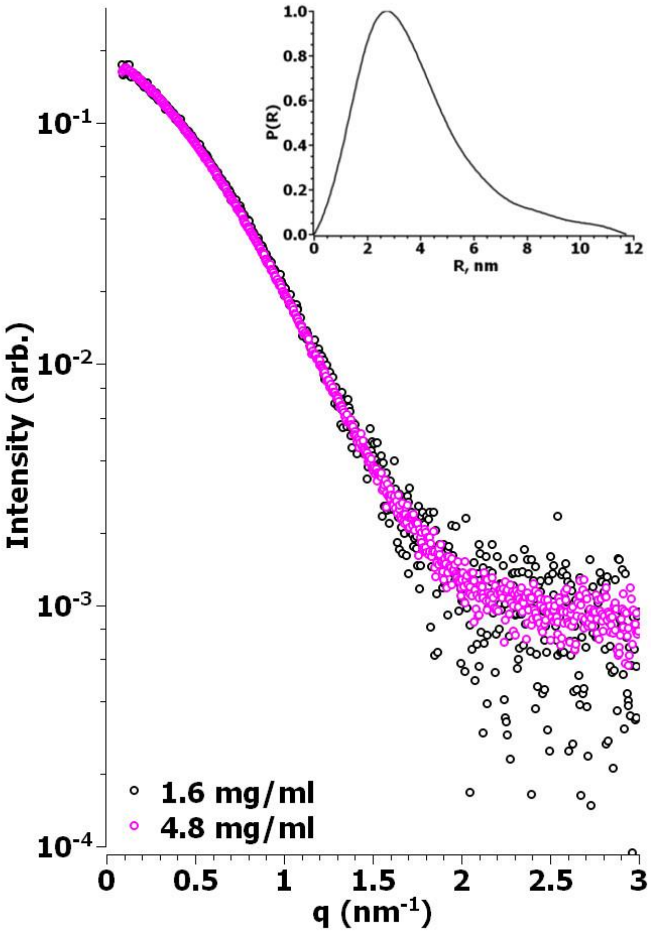

Figure S7: SAXS intensities vs. scattering vector **q** at two different AnPrx6 concentrations. The SAXS intensity data for AnPrx6 at 1.6 mg/ml (black circles) and 4.8 mg/ml (magenta circles), were scaled to visualize the lack of concentration dependent protein-protein interactions. Due to the low noise level, the actual experimental data up to 3 nm^{-1} displayed instead of the fitted curves. The inset shows the pair-distance distribution function calculated using Primus.

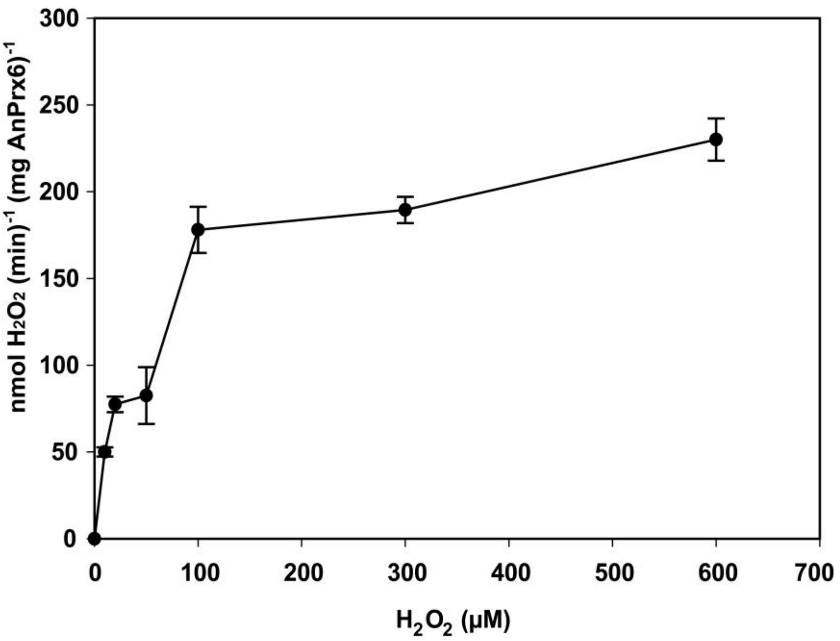

Figure S8. The AnPrx6 peroxidase activity was measured at increasing concentrations of H_2O_2 in the range from 0 µM to 600 µM. All measurements were carried out in triplicates. The average value is marked by a dot and the error bars mark the uncertainty of the measurements.

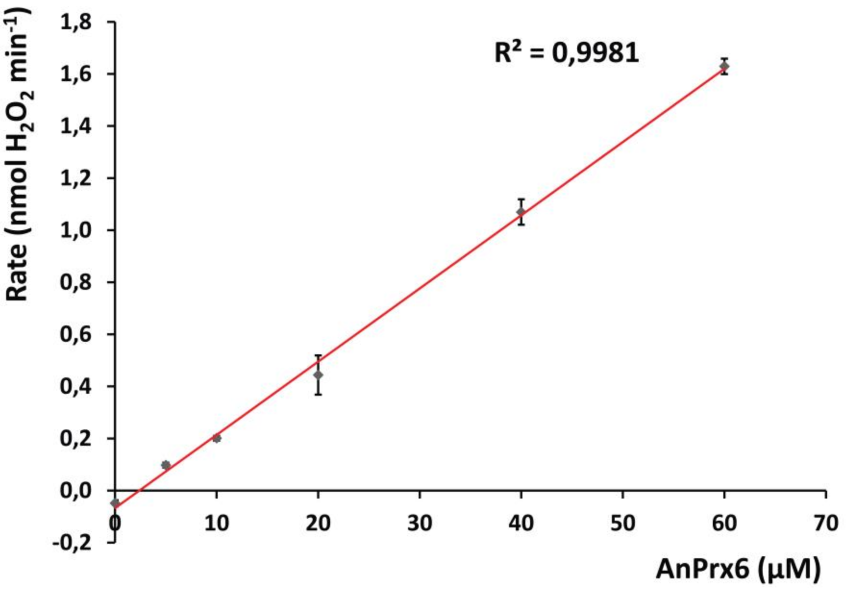

Figure S9. H_2O_2 Consumption rate vs. AnPrx6 concentration. The graph shows a linear dependency of the H_2O_2 consumption rate with respect to the enzyme concentration, which suggests that no structural changes, such as oligomerization, occur as the protein concentration increases from 0 µM to 60 µM. The error for the first three points is very small and which results in error bars that are not clearly visible.

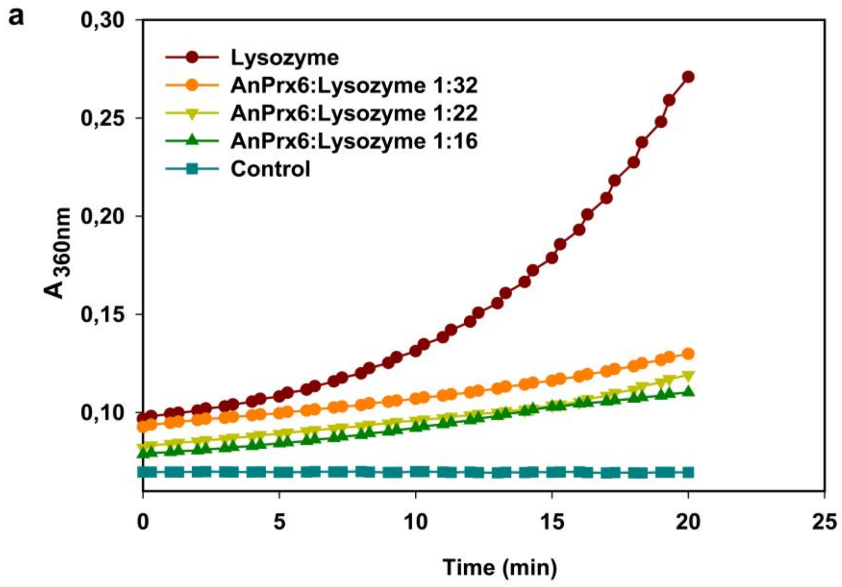

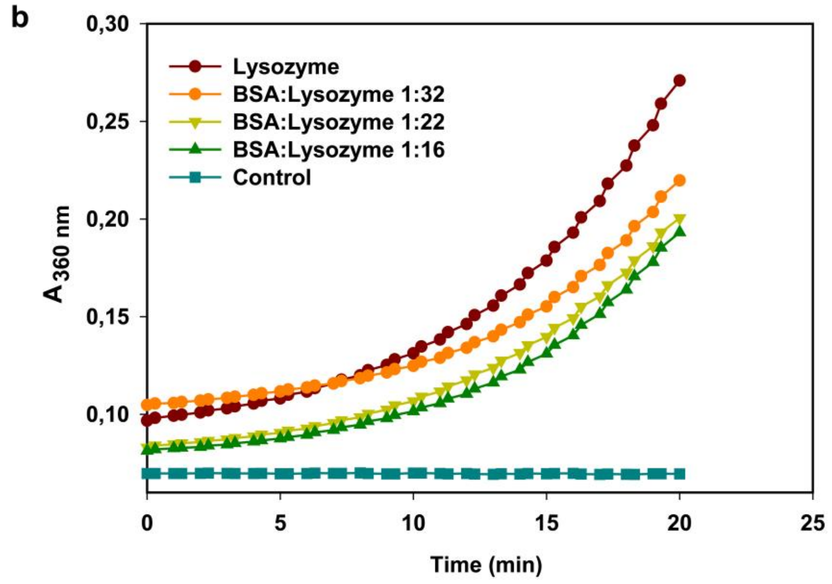

Figure S10. Chaperone activity assays. AnPrx6 prevents the thermal aggregation of hen egg white Lysozyme at 43°C. (**a**) Aggregation of Lysozyme at decreasing molar AnPrx6 to Lysozyme ratios of 1:16, 1:22, and 1:32. (**b**) Control experiment showing the aggregation of lysozyme where AnPrx6 was replaced by BSA. The aggregation of lysozyme was monitored by measuring the degree of light scattered at 360 nm. To determine the baseline level of scattering (negative control) only buffer and DTT and was used.

Supplementary References:

1 Mishra, Y., Chaurasia, N. & Rai, L. C. AhpC (alkyl hydroperoxide reductase) from Anabaena sp. PCC 7120 protects Escherichia coli from multiple abiotic stresses. *Biochem. Biophys. Res. Commun.* **381,** 606-611 (2009).

2 Mishra, Y. *et al.* Expression, purification, crystallization and preliminary X-ray crystallographic studies of alkyl hydroperoxide reductase (AhpC) from the cyanobacterium Anabaena sp. PCC 7120. *Acta Crystallogr. Sect. F Struct. Biol. Cryst. Commun.* **67,** 1203-1206 (2011).

3 Gasteiger, E. *et al.* ExPASy: The proteomics server for in-depth protein knowledge and analysis. *Nucleic Acids Res.* **31,** 3784-3788 (2003).

4 Abagyan, R. T., M.; Kuznetsov, D. ICM - A new method for protein modeling and design: Applications to docking and structure prediction from the distorted native conformation. *J. Comp. Chem.* **15,** 488–506 (1994).

5 Abagyan, R. A. & Batalov, S. Do aligned sequences share the same fold? *J. Mol. Biol.* **273,** 355-368 (1997).

6 Choi, H. J., Kang, S. W., Yang, C. H., Rhee, S. G. & Ryu, S. E. Crystal structure of a novel human peroxidase enzyme at 2.0 A resolution. *Nat. Struct. Biol.* **5,** 400-406 (1998).

7 Smeets, A. *et al.* The crystal structure of the C45S mutant of annelid Arenicola marina peroxiredoxin 6 supports its assignment to the mechanistically typical 2-Cys subfamily without any formation of toroid-shaped decamers. *Protein Sci.* **17,** 700-710 (2008).

8 Vedadi, M. *et al.* Genome-scale protein expression and structural biology of Plasmodium falciparum and related Apicomplexan organisms. *Mol. Biochem. Parasitol.* **151,** 100-110 (2007).

9 Mizohata, E. *et al.* Crystal structure of an archaeal peroxiredoxin from the aerobic hyperthermophilic crenarchaeon Aeropyrum pernix K1. *J. Mol. Biol.* **354,** 317-329 (2005).

10 Nakamura, T. *et al.* Structure of peroxiredoxin from the anaerobic hyperthermophilic archaeon Pyrococcus horikoshii. *Acta Crystallogr. Sect. F Struct. Biol. Cryst. Commun.* **69,** 719-722 (2013).

11 Pineyro, M. D. *et al.* Crystal structure of the tryparedoxin peroxidase from the human parasite Trypanosoma cruzi. *J. Struct. Biol.* **150,** 11-22 (2005).

12 Hirotsu, S. *et al.* Crystal structure of a multifunctional 2-Cys peroxiredoxin heme-binding protein 23 kDa/proliferation-associated gene product. *Proc. Natl. Acad. Sci. U. S. A.* **96,** 12333-12338 (1999).

13 Fiorillo, A., Colotti, G., Boffi, A., Baiocco, P. & Ilari, A. The crystal structures of the tryparedoxin-tryparedoxin peroxidase couple unveil the structural determinants of Leishmania detoxification pathway. *PLoS Negl. Trop. Dis.* **6,** e1781 (2012).

14 Alphey, M. S., Bond, C. S., Tetaud, E., Fairlamb, A. H. & Hunter, W. N. The structure of reduced tryparedoxin peroxidase reveals a decamer and insight into reactivity of 2Cys-peroxiredoxins. *J. Mol. Biol.* **300,** 903-916 (2000).

15 Schroder, E. *et al.* Crystal structure of decameric 2-Cys peroxiredoxin from human erythrocytes at 1.7 A resolution. *Structure* **8,** 605-615 (2000).

16 Saccoccia, F. *et al.* Moonlighting by different stressors: crystal structure of the chaperone species of a 2-Cys peroxiredoxin. *Structure* **20,** 429-439 (2012).

17 Nguyen, J. B. *et al.* Peroxiredoxin-1 from the human hookworm Ancylostoma ceylanicum forms a stable oxidized decamer and is covalently inhibited by conoidin A. *Chem. Biol.* **20,** 991-1001 (2013).

18 Papinutto, E. *et al.* Crystal structure of alkyl hydroperoxide-reductase (AhpC) from Helicobacter pylori. *Biochim. Biophys. Acta* **1753,** 240-246 (2005).
19 McNicholas, S., Potterton, E., Wilson, K. S. & Noble, M. E. Presenting your structures: the CCP4mg molecular-graphics software. *Acta Crystallogr. D Biol. Crystallogr.* **67,** 386-394 (2011).
20 Labrador, A., Cerenius, Y., Svensson, C., Theodor, K. & Plivelic, T. The yellow minihutch for SAXS experiments at MAX IV Laboratory. *J. Phys. Conf. Ser.* **425** (2013).
21 Petoukhov, M. V. *et al.* New developments in the ATSAS program package for small-angle scattering data analysis. *J. Appl. Crystallogr.* **45,** 342-350 (2012).
22 Konarev, P. V., Volkov, V. V., Sokolova, A. V., Koch, M. H. J. & Svergun, D. I. PRIMUS: A Windows PC-based system for small-angle scattering data analysis. *J. Appl. Crystallogr.* **36,** 1277-1282 (2003).
23 Porod, G. in *Small Angle X-Ray Scattering* (eds Glatter O. & Kratky O.) Ch. 2, 17-51 (Akademic Press Inc. (London) LTD, 1982).
24 Jiang, Z. Y., Woollard, A. C. & Wolff, S. P. Hydrogen peroxide production during experimental protein glycation. *FEBS Lett.* **268,** 69-71 (1990).
25 Flohe, L. *et al.* Tryparedoxin peroxidase of Leishmania donovani: molecular cloning, heterologous expression, specificity, and catalytic mechanism. *Arch. Biochem. Biophys.* **397,** 324-335 (2002).
26 Poole, L. B., Hall, A. & Nelson, K. J. Overview of peroxiredoxins in oxidant defense and redox regulation. *Curr. Protoc. Toxicol.* **Chapter 7,** Unit7 9 (2011).
27 Wood, Z. A., Poole, L. B., Hantgan, R. R. & Karplus, P. A. Dimers to doughnuts: redox-sensitive oligomerization of 2-cysteine peroxiredoxins. *Biochemistry* **41,** 5493-5504 (2002).
