# Supplementary figures and images for: Active-site plasticity revealed in the asymmetric dimer of AnPrx6 the 1-Cys peroxiredoxin and molecular chaperone from Anabaena sp. PCC 7120
Source: Sci Rep. 2017 Dec 7;7:17151. doi: 10.1038/s41598-017-17044-3 (PMC5719442; doi:10.1038/s41598-017-17044-3)

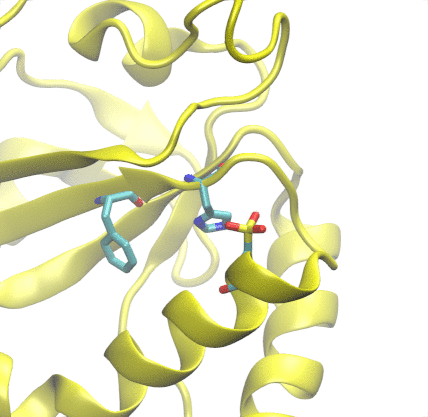

Supplement: Supplementary file 2 — Supplementary simulation Sim1 [file 41598_2017_17044_MOESM2_ESM.gif]

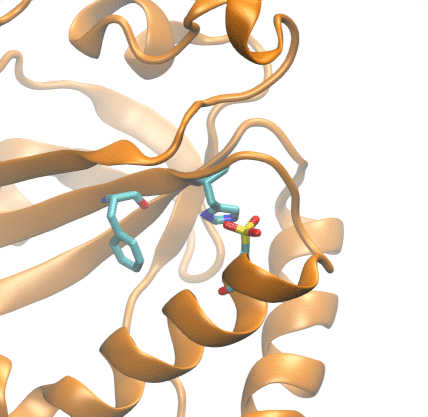

Supplement: Supplementary file 3 — Supplementary simulation Sim3 [file 41598_2017_17044_MOESM3_ESM.gif]
